# Ameliorative Effects of Schisandrol A on Lipopolysaccharide-Induced Intestinal Injury in Mice

**DOI:** 10.3390/nu18172808

**Published:** 2026-08-27

**Authors:** Xue Wang, Renying Wang, Yiwen Wang, Nanqi Zhang, Wanying Li, Mingjie Song, Yingping Wang

**Affiliations:** State Local Joint Engineering Research Center of Ginsesgtareeding and Application, College of Traditional Chinese Medicine, Jilin Agricultural University, Changchun 130118, China; 13841305033@163.com (X.W.); wry970821@163.com (R.W.);

**Keywords:** Schisandrol A, lipopolysaccharide, intestinal injury, 16S rRNA, serum metabolomics

## Abstract

*Schisandra chinensis* (Turcz.) Baill. is a traditional medicinal and edible plant abundant in bioactive constituents which has been proven to exert prominent intestinal protective effects. Schisandrol A (SA) is one of the lignan components with a relatively high content in this plant. This study aims to evaluate the intestinal protective effects of schisandrol A (SA) in a lipopolysaccharide (LPS)-induced intestinal injury mouse model. An LPS-induced mouse model was established and treated with SA in different groups, and oxidative stress, inflammatory markers, apoptosis, NF-κB signaling pathway proteins, and tight junction proteins were examined. Meanwhile, 16S rRNA gene sequencing and serum metabolomics were employed to evaluate alterations in the gut microbiota and host serum biochemical profiles. The results showed that SA significantly alleviated LPS-induced intestinal injury. SA attenuated oxidative stress, upregulated the anti-inflammatory cytokine IL-10, and reduced the levels of the pro-inflammatory cytokines TNF-α, IL-6, and IL-1β. In addition, SA inhibited apoptosis and the activation of the NF-κB pathway, while upregulating the expression of the tight junction proteins ZO-1 and occludin. Gut microbiota analysis revealed that SA reversed the LPS-induced gut microbiota dysbiosis. Serum metabolomics indicated that SA exerted its intestinal protective effects by targeting and modulating several core host serum biochemical pathways. In conclusion, SA reduces oxidative stress and inflammatory responses, restores the intestinal barrier, reshapes the gut microbiota, improves disturbed serum biochemical profiles, and inhibits the activation of the NF-κB pathway, thereby effectively ameliorating intestinal injury in mice.

## 1. Introduction

Intestinal injury is a critical clinical condition frequently resulting in intra-abdominal infection [1]. It primarily affects the small intestine, followed by the colon, and is characterized by severe abdominal pain, diarrhea, and hematochezia [2]. As a life-threatening clinical emergency, this condition frequently leads to severe complications such as intestinal adhesions, perforation, and fistula formation, which significantly increase patient mortality and severely diminish their quality of life. Pathologically, the condition involves oxidative stress, inflammatory cascades, epithelial cell damage, and gut dysbiosis [3,4,5,6,7]. Currently, antibiotics used for treating intestinal injury in clinical practice often have numerous side effects and can easily lead to gut microbiota dysbiosis. This dysbiosis in turn exacerbates mucosal barrier disruption and inflammatory responses, further aggravating intestinal damage. Therefore, exploring safe and effective natural compounds that can synergistically repair the intestinal barrier, modulate the gut microbiota, and exert anti-inflammatory effects holds significant research value for the prevention and amelioration of intestinal injury.

The dried ripe fruit of *Schisandra chinensis* is a commonly used medicinal material belonging to the Magnoliaceae family [8]. Historically documented in the “Shennong Bencao Jing” (an ancient Chinese pharmacopoeia) and “Bencao Bei Yao” (a classic Chinese herbal medicine treatise), it is recognized for its kidney-tonifying, heart-calming, and astringent properties [9,10]. Modern phytochemical studies have revealed that *Schisandra chinensis* is rich in active constituents, including lignans, terpenoids, polysaccharides, and volatile oils [11,12]. These constituents exhibit distinct pharmacological properties. For instance, polysaccharides are well known for their immunomodulatory effects [13], whereas volatile oils mainly contribute to antibacterial activity [14]. Notably, lignans, the major stable components in most extracts, have been widely reported to exert potent antioxidant, anti-inflammatory, and hepatoprotective effects [15,16]. Schisandrol A (SA) is one of the signature lignans of Schisandra chinensis [17], and exhibits potent hepatoprotective, anti-inflammatory, antioxidant, and anti-apoptotic properties [18,19,20,21]. In recent years, the potential of Schisandra extracts to repair intestinal damage has garnered considerable attention [22,23,24].

Lipopolysaccharide (LPS), a primary component of the outer membrane of Gram-negative bacteria, is a potent inducer of systemic inflammatory response syndrome (SIRS) [25] and intestinal endotoxemia [26]. Due to its ability to trigger robust inflammatory responses and cellular apoptosis, the LPS-induced model is widely utilized to simulate severe intestinal hyperpermeability and gut barrier dysfunction [27,28,29].

Preliminary observations within our research group indicated that schisandrol A could maintain intestinal tissue integrity, restore microbial balance and alleviate diarrheal symptoms in mice. Nevertheless, its effects and underlying mechanisms against intestinal injury remain unclear. Therefore, this study employed an LPS-induced intestinal injury model in mice to investigate the therapeutic effects and mechanisms of *Schisandra chinensis* extract. Combined with serum metabolomics analysis, the potential pathways were further revealed, which is intended to lay a scientific and theoretical foundation for the development of *Schisandra chinensis* extract products with intestinal health-improving effects.

## 2. Materials and Methods

### 2.1. Reagents and Kits

Schisandrol A (HPLC > 98%, batch number MUST-22052011) was purchased from Chengdu Mansite Biotechnology Co., Ltd., Chengdu, China. Lipopolysaccharide (LPS, batch number 0000189842) was purchased from Merck Sigma Chemical Company, St. Louis, MO, USA. The reactive oxygen species (ROS) kit (batch number S0033S) and bicinchoninic acid (BCA) protein content detection kit (batch number 020123230421) were purchased from Biyun Tian Biotechnology, Beijing, China. The diamine oxidase (DAO) kit (batch number 20240712) was purchased from Nanjing Jiancheng Bioengineering Laboratory, Nanjing, China. Cell lysate (batch number 2311006) and protease inhibitor (batch number 20230830) were purchased from Beijing Soleibao Technology Co., Ltd., Beijing, China. The malondialdehyde (MDA) kit (batch number 20231223), glutathione (GSH) kit (batch number 20230603), superoxide dismutase (SOD) kit (batch number 20230503), and lactate dehydrogenase (LDH) kit (batch number 20230712) were purchased from Nanjing Jiancheng Bioengineering Laboratory. Absolute ethanol and xylene were purchased from Sinopharm Chemical Reagent Co., Ltd. (Shanghai, China). Paraffin was purchased from Leica Biosystems (Wetzlar, Germany).

### 2.2. Main Instruments

A full-wavelength microplate reader (Thermo Fisher Scientific, Waltham, MA, USA, model 1510) was used for enzyme-linked immunosorbent assay (ELISA). A high-speed freeze centrifuge (Beijing Dalong Xingchuang Experimental Instrument Co., Ltd., Beijing, China, model D1524R) and an electrophoretic apparatus (Shanghai Tianneng Technology Co., Ltd., Shanghai, China, model EPS 300) were also used in this study. An optical microscope (Olympus BX53, Olympus Corporation, Tokyo, Japan) was used for observing and photographing the stained tissue sections.

### 2.3. Experimental Animals and Grouping

BALB/c mice were purchased from Beijing Huafukang Biotechnology Co., Ltd., Beijing, China (license number: SCXK (Beijing) 2019-0008) and housed in the specific pathogen-free (SPF) animal facility of the Experimental Animal Center, Jilin Agricultural University. The feeding conditions were controlled as follows: room temperature of 21–23 °C, relative humidity of 55–67%, and a 12 h light/dark cycle. Mice had free access to food and water throughout the experiment. The experimental protocol was approved by the Animal Experiment Ethics Committee of Jilin Agricultural University, and both animal welfare and experimental procedures complied with the committee’s regulations (Animal Experimentation Ethics Approval Number: 20230509002).

**Figure 1:**
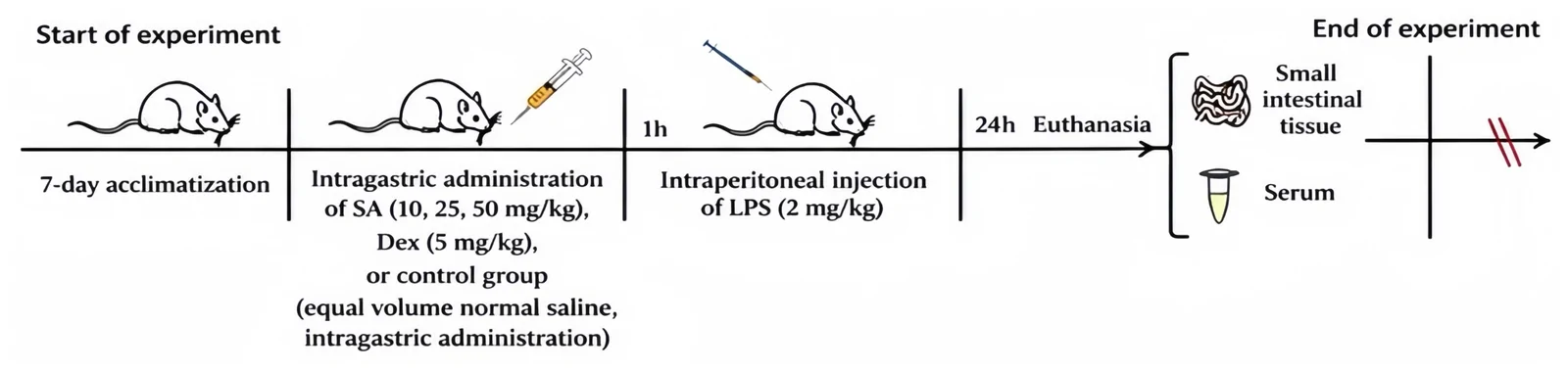
Experimental design of schisandrol A effectively improving lipopolysaccharide-induced intestinal injury in mice.

According to the experimental design shown in Figure 1, sixty 9-week-old male BALB/c mice were acclimatized for one week and then randomly divided into six groups using a computer-generated random number table (*n* = 10 per group): normal control (Con) group, model (LPS) group, positive drug dexamethasone (Dex) group, and SA high-, medium-, and low-dose groups (50 mg/kg, 25 mg/kg, and 10 mg/kg, respectively). The group size (*n* = 10) was determined based on previously published similar in vivo studies and our preliminary experimental data, which was sufficient to observe significant statistical trends in this exploratory study. As a prophylactic treatment, mice in the SA groups were administered intragastrically with SA dissolved in olive oil at the corresponding doses, while those in the Con and LPS groups received an equal volume of olive oil intragastrically. Mice in the Dex group were intraperitoneally injected with 5 mg/kg Dex based on previous studies [30]. One hour after these treatments, mice in the SA groups (high, medium, and low doses), the Dex group, and the LPS group were intraperitoneally injected with 2 mg/kg LPS dissolved in normal saline to establish the acute inflammatory model. The Con group was injected with an equal volume of normal saline instead. Twenty-four hours after model establishment [31], all animals were euthanized, and blood samples were collected from the orbital venous plexus. The collected blood was centrifuged at 4000 rpm for 20 min to obtain fresh serum, which was reserved for subsequent experiments. Meanwhile, small intestinal tissues (duodenum, ileum) were dissected and rinsed with normal saline. A portion of the tissues was fixed in 4% paraformaldehyde for histopathological staining, and the remaining small intestinal tissues were snap-frozen in liquid nitrogen and stored long-term at −80 °C. The experimental schematic is shown in Figure 1.

### 2.4. Macroscopic Damage to Small Intestine Tissue

After washing the small intestinal tissue with cold physiological saline, the tissue was placed flat on a clean white background plate to observe macroscopic damage. Photographs of the tissues were taken for visual documentation using a smartphone camera. Additionally, body weight changes in mice were recorded before and after model establishment.

### 2.5. Detection of Diamine Oxidase (DAO) Activity in Mouse Serum

Mouse serum samples were collected and processed according to the kit instructions. The activity of diamine oxidase (DAO) in mouse serum was measured using an enzyme-linked immunosorbent assay (ELISA) reader to reflect the degree of intestinal injury and repair.

### 2.6. Intestinal Hematoxylin and Eosin (HE) Stained Sections

The duodenum and ileum of mice from each treatment group were dissected, and segments approximately 0.5 cm in length were fixed in 4% paraformaldehyde for 24 h, and then dehydrated in graded concentrations of ethanol (70%, 80%, 90%, 95%, and 100%). After clearing with graded concentrations of xylene (ethanol:xylene = 1:1, followed by pure xylene), the tissues were embedded in paraffin, sectioned, and stained according to the instructions of the hematoxylin and eosin (HE) staining kit. The stained sections were observed and photographed under a microscope (200×). To evaluate the histopathological injury, we graded the tissue changes according to previously established criteria [32,33]. This scoring system assessed crypt structural damage, inflammatory cell infiltration, epithelial shedding, and villus/mucosal architectural destruction. The histopathological injury was semi-quantitatively evaluated based on the scoring criteria in Table 1.

### 2.7. Immunohistochemistry

Paraffin sections were deparaffinized, rehydrated, and subjected to citrate-buffered antigen retrieval. Endogenous peroxidase activity was blocked with catalase. Sections were incubated overnight at 4 °C with primary antibodies against ZO-1 (1:500–1:1000) and occludin (1:500–1:1000). On the following day, sections were incubated with secondary antibodies, followed by 3,3′-diaminobenzidine (DAB) chromogenic detection and hematoxylin counterstaining. Images were acquired using a digital whole-slide scanner (Pannoramic MIDI, 3DHISTECH, Budapest, Hungary). After scanning, digital images were viewed and exported using CaseViewer 2.4 software.

### 2.8. Detection of Inflammatory Factors in Serum

According to the manufacturer’s instructions, the levels of TNF-α, IL-6, IL-10, and IL-1β in mouse serum were measured using ELISA kits.

### 2.9. Measurement of Oxidative Markers in Small Intestine Tissue

Small intestinal tissues were collected from each group of mice, rinsed with ice-cold normal saline, and excess water was blotted dry. The tissues were then snap-frozen in liquid nitrogen and stored in a −80 °C freezer for subsequent use. Before analysis, 0.5 g of small intestinal tissue from each treatment group was weighed and placed into a glass homogenizer tube, followed by the addition of 4.5 mL of ice-cold normal saline. The tissue was homogenized, and the homogenate was centrifuged at 3500× *g* for 10 min at 4 °C to obtain a 10% tissue homogenate. The contents of LDH, MDA, GSH, and SOD in the intestinal tissue homogenate were detected according to the kit instructions.

### 2.10. Western Blot

Small intestine tissues (20 mg) were lysed in 400 μL of lysis buffer, centrifuged at 10,000 rpm for 15 min at 4 °C, and the supernatant was collected. Protein concentration was determined using a BCA assay. Samples were mixed with 5× loading buffer (4:1 ratio) and denatured at 95 °C for 10 min.

Proteins were separated by sodium dodecyl sulfate-polyacrylamide gel electrophoresis (SDS-PAGE), transferred to polyvinylidene fluoride (PVDF) membranes, and blocked with rapid blocking buffer for 10 min. Membranes were then incubated with primary antibodies: Bax, Bcl-2, Caspase-3, Cleaved caspase-3, Cytochrome C, NF-κB p65, P-p65, IκBα, P-IκBα, IKKα, IKKβ (all 1:1000), and β-actin (1:5000). After washing with Tris-buffered saline containing Tween-20 (TBST), membranes were incubated with horseradish peroxidase (HRP)-conjugated secondary antibodies (1:5000–1:10,000) for 1.5 h at room temperature. Protein bands were visualized using enhanced chemiluminescence (ECL) chemiluminescence and quantified using ImageJ software (version 1.54v).

### 2.11. Immunofluorescence Assay

The expression and localization of p65 protein in mouse ileum tissues were examined by immunofluorescence staining. Briefly, paraffin-embedded sections were deparaffinized, rehydrated, and subjected to antigen retrieval using citrate buffer. After blocking with 5% bovine serum albumin (BSA) for 1 h at room temperature, the sections were incubated overnight at 4 °C with the primary antibody against NF-κB p65 (dilution 1:200). Subsequently, the sections were incubated with a fluorescence-conjugated secondary antibody for 1 h at room temperature in the dark. The nuclei were counterstained with 4′,6-diamidino-2-phenylindole (DAPI). The immunofluorescence images were captured using a fluorescence microscope (Nikon Eclipse C1, Nikon Corporation, Tokyo, Japan) equipped with a digital camera, and the whole slide images were scanned using a digital slide scanner (LG-FS80, Servicebio, Wuhan, China; or Pannoramic MIDI, 3DHISTECH, Budapest, Hungary), and the fluorescence intensity was analyzed using ImageJ software (version 1.54v).

### 2.12. 16S rRNA Sequencing Analysis

After dissection, the cecal segments were quickly snap-frozen in liquid nitrogen and then transferred to a −80 °C freezer. Total microbial genomic DNA was extracted from cecal contents, and the V3–V4 hypervariable region of the bacterial 16S rRNA gene was amplified by PCR. Sequencing was performed on the Illumina platform, and raw reads were quality-filtered, clustered into amplicon sequence variants (ASVs). Taxonomic annotation, alpha diversity, beta diversity, and differential microbiota analysis were conducted. Alpha diversity indices, including Chao1 and observed ASVs reflecting species richness, were calculated to evaluate the species richness of gut microbiota. Principal coordinate analysis (PCoA) based on Bray–Curtis distance was applied to characterize the overall structural variation of the gut microbiome among groups. All sequencing and bioinformatic analyses were completed by Beijing Baimaike Biotechnology Co., Ltd., Beijing, China.

### 2.13. Serum Metabolomics

Mouse blood samples were collected from the orbital venous plexus of mice. After standing at room temperature for 30 min, samples were centrifuged at 3000× *g* for 15 min at 4 °C. The supernatant serum was harvested, aliquoted, and stored at −80 °C prior to metabolomic analysis, which was performed by Beijing Baimaike Biotechnology Co., Ltd. [34].

The detection platform consisted of a Waters Acquity I-Class PLUS ultra-high-performance liquid chromatography (UPLC) system coupled to a Waters Xevo G2-XS QTOF high-resolution mass spectrometer (Waters, Milford, MA, USA). Sample detection and analysis were conducted according to the manufacturer’s standard parameters.

### 2.14. Statistical Analysis

All numerical data collected are uniformly expressed as the mean ± standard deviation (SD). The sample size, denoted by “*n*” (number of animals or replicates), is precisely noted for each dataset. Sample sizes were selected with reference to previously published similar in vivo studies. The Shapiro–Wilk test was applied to examine the normality of data distribution. Statistical significance was evaluated using one-way analysis of variance (one-way ANOVA), followed by Dunnett’s post hoc test to determine differences between the control group and the other experimental groups. All computations were performed using GraphPad Prism software (Version 9.0, GraphPad Software, Boston, MA, USA). A probability (*p*) value set below 0.05 (*p* < 0.05) was deemed statistically significant.

## 3. Results

### 3.1. SA Reduces the Degree of Intestinal Injury in Mice

**Figure 2:**
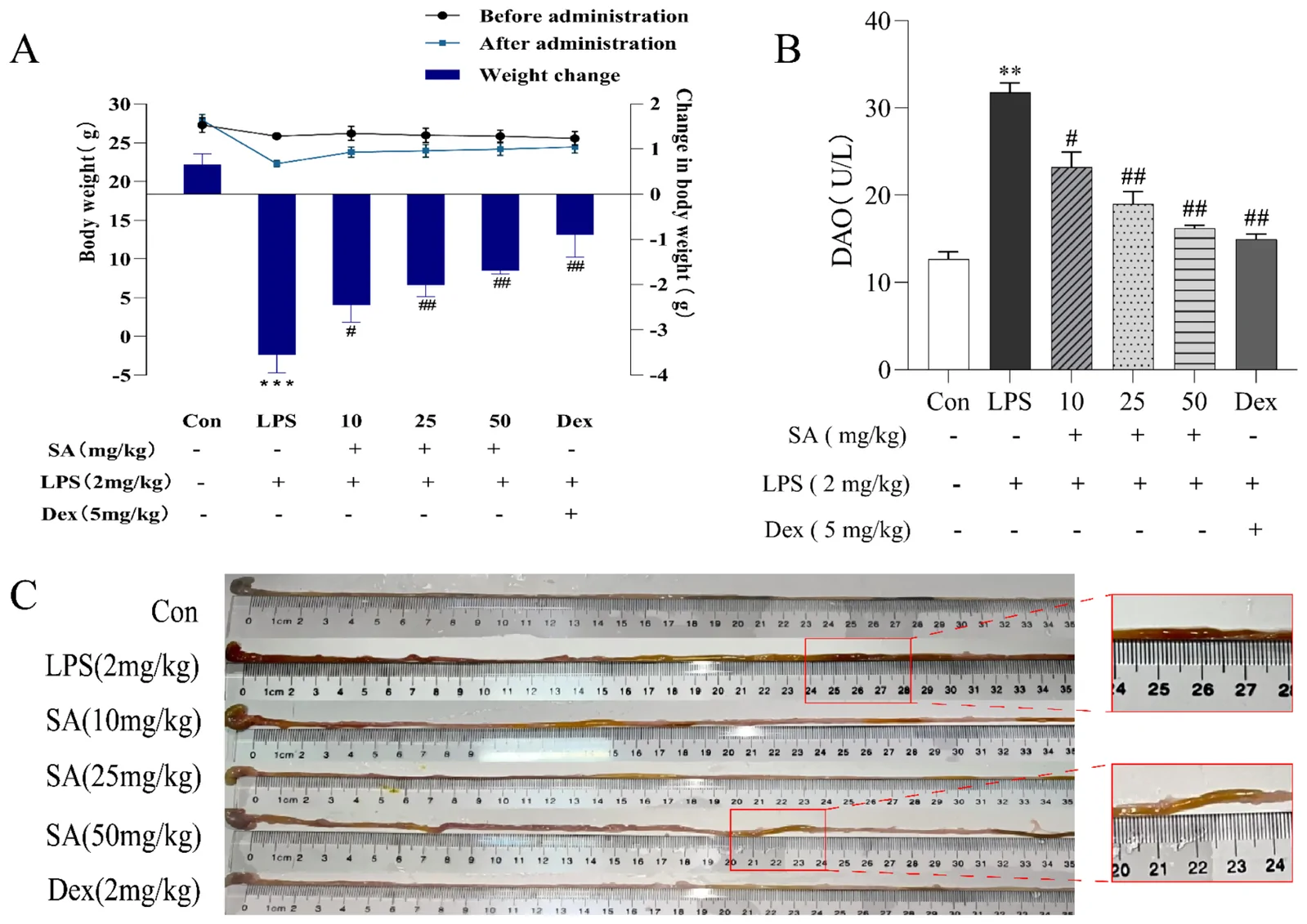
The protective effects of SA on intestinal injury in mice. (**A**) Intestine tissue of mice. (**B**) Serum DAO level in mice of each group. (**C**) Macroscopic condition of mouse small intestine tissue. Symbols “−” and “+” indicate absence and treatment with the corresponding agent, respectively. Data are expressed as mean ± SD, *n* = 10. * *p* < 0.05, ** *p* < 0.01, *** *p* < 0.001 versus Con group; # *p* < 0.05, ## *p* < 0.01, ### *p* < 0.001 versus LPS group.

Twenty-four hours after LPS injection, mice in the LPS group exhibited more severe symptoms compared with those in the SA treatment groups, including ruffled fur, mental fatigue, slow movement, increased sleep duration, and noticeable secretions from the corners of the eyes. As shown in Figure 2A, the body weight of mice in the LPS group was significantly reduced, and SA administration ameliorated this LPS-induced weight loss. DAO is a highly active intracellular enzyme in the villi of the mammalian small intestinal mucosa, and it can reflect the integrity and degree of damage to the intestinal mechanical barrier [35]. As illustrated in Figure 2B, DAO activity was significantly increased in the LPS group compared to the Con group, confirming the successful establishment of the intestinal injury model. SA treatment ameliorated this abnormality. It can also be observed in Figure 2C that, compared with the Con group, the small intestinal tissue of the LPS group appeared reddened and showed obvious hyperemia. In contrast, following SA treatment, the small intestinal hyperemia in mice was not obvious, and the appearance tended towards that of the Con group in a dose-dependent manner. These results collectively demonstrate that SA significantly alleviated the degree of intestinal damage in mice.

### 3.2. SA Alleviates Intestinal Barrier Damage in Mice

**Figure 3:**
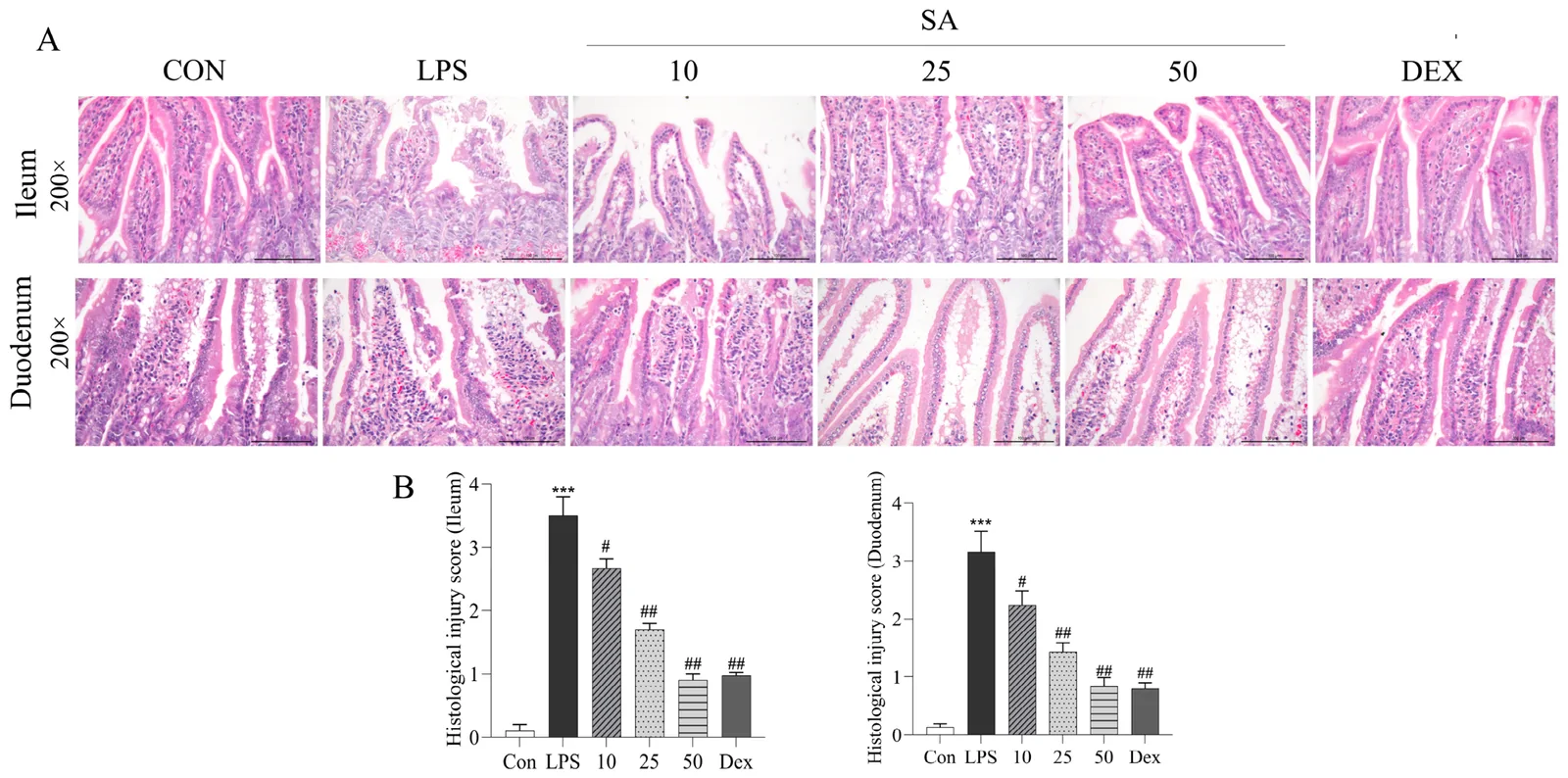
The protective effect of SA on the intestinal barrier in mice. (**A**) Representative histopathological images of the ileum and duodenum (HE staining, ×200). (**B**) Quantitative analysis of the histological injury scores in the ileum and duodenum. Data are expressed as mean ± SD (*n* = 3). ** *p* < 0.01, *** *p* < 0.001 versus Con group; # *p* < 0.05, ## *p* < 0.01, ### *p* < 0.001 versus LPS group.

To evaluate the ameliorative effect of SA on LPS-induced small intestinal injury, the degrees of damage in the duodenum and ileum of mice were observed and compared among groups. As shown in Figure 3, the LPS group exhibited epithelial cell necrosis, exposed lamina propria, and significantly shortened, narrowed and atrophied intestinal villi. In the SA treatment groups, the symptoms of intestinal villus breakage and mucosal ulceration were alleviated. In the SA treatment groups, the symptoms of intestinal villus breakage and mucosal ulceration were alleviated, and the degree of small intestinal tissue damage was reduced compared with that in the LPS group. Moreover, the morphology of the SA (50 mg/kg) group and the Dex group approached that of the Con group. These observations indicate that following SA intervention, the small intestinal tissue of mice with intestinal injury indeed showed significant damage and subsequent improvement, with the ileum exhibiting more pronounced injury and a better therapeutic response to SA. Therefore, the ileum was selected for subsequent experiments.

**Figure 4:**
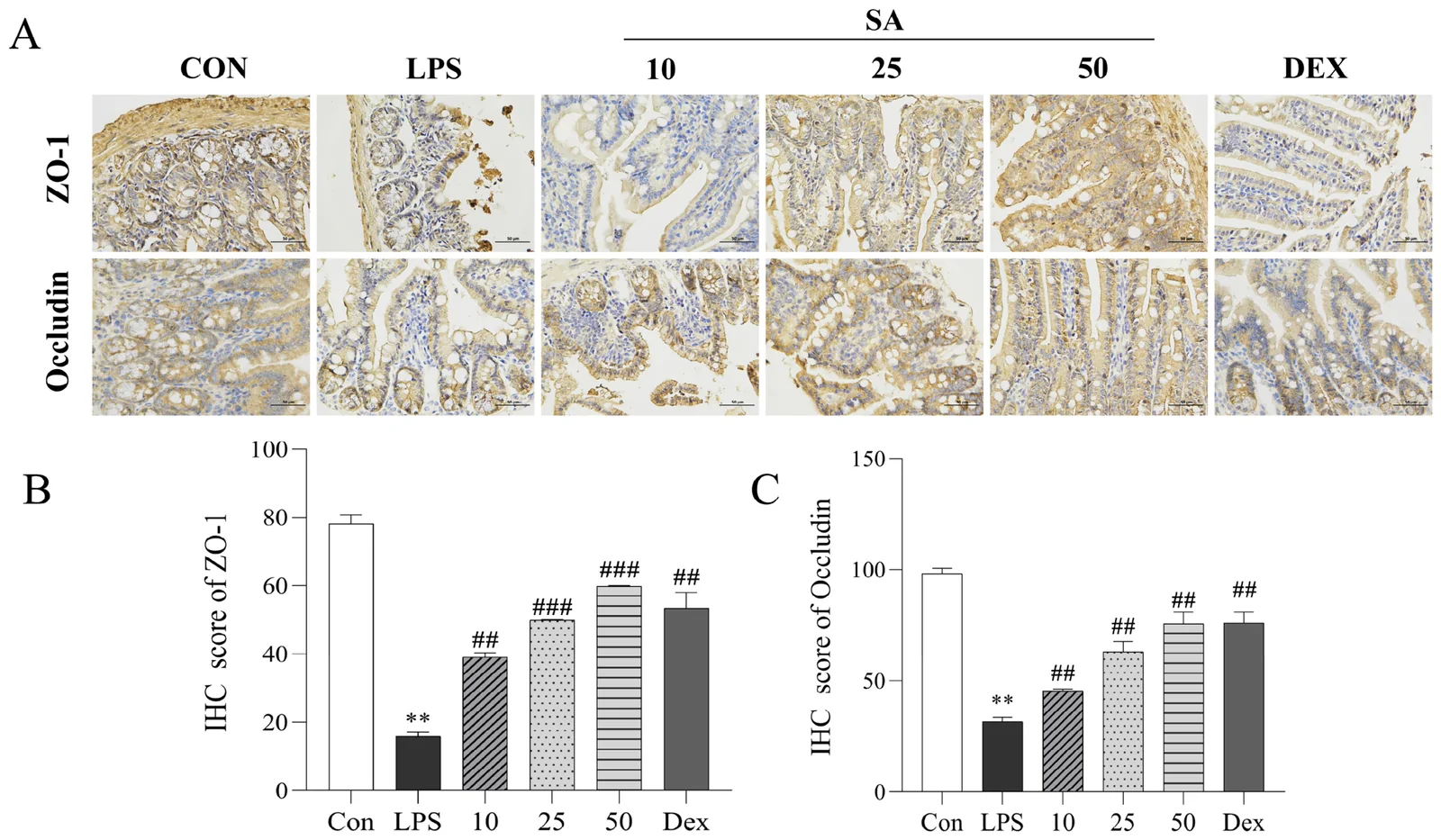
Immunohistochemical detection of tight junction proteins ZO-1 and occludin in colon. (**A**) Typical IHC micrographs of ZO-1 and occludin (IHC, ×400). (**B**,**C**) Semi-quantitative IHC scores of ZO-1 and occludin. ** *p* < 0.01 vs. Con; ## *p* < 0.01, ### *p* < 0.001 vs. LPS. Con, control; LPS, LPS-induced model; 10, 25, 50 mg/kg SA; Dex, dexamethasone.

Tight junction proteins ZO-1 and occludin play critical roles in maintaining the protective function of the intestinal barrier. Immunohistochemistry was used to analyze the regulatory effect of SA on the expression of tight junction proteins in mouse ileal tissue. As shown in Figure 4, SA alleviated the LPS-induced decrease in ZO-1 and occludin protein expression. These results suggest that SA can ameliorate LPS-induced intestinal barrier damage in mice.

### 3.3. SA Regulates Intestinal Oxidation, Apoptosis, and Inflammation in Mice

**Figure 5:**
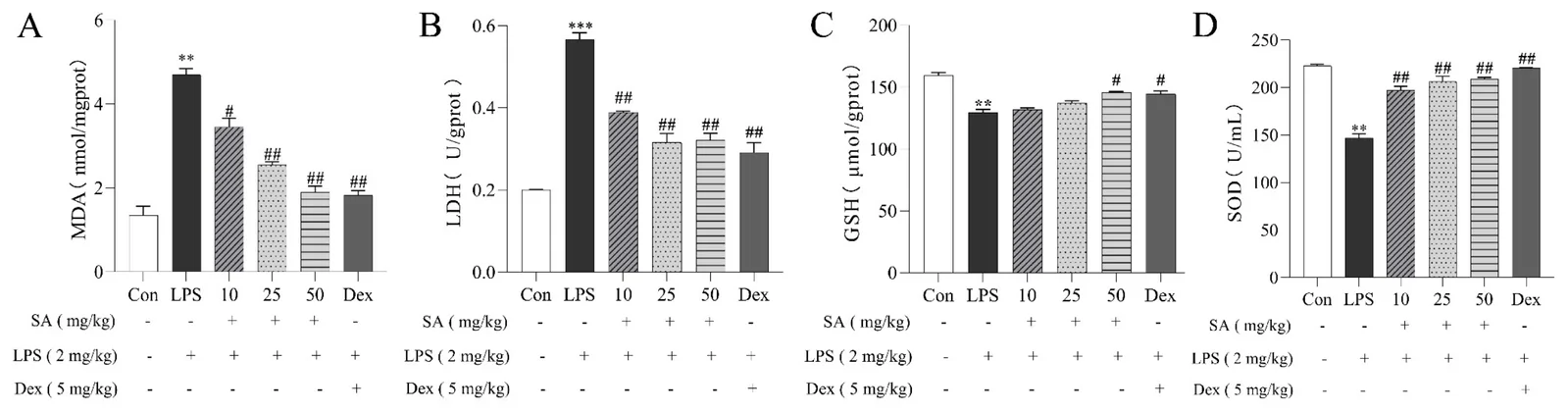
SA regulated oxidative stress indicators in the colon tissues of LPS-challenged mice. (**A**) The level of MDA in mouse ileum tissue. (**B**) The level of LDH in mouse ileum tissue. (**C**) The level of GSH in mouse ileum tissue. (**D**) The level of SOD in mouse ileum tissue. Symbols “−” and “+” indicate absence and treatment with the corresponding agent, respectively. Data are expressed as mean ± SD, *n* = 3. * *p* < 0.05, ** *p* < 0.01, *** *p* < 0.001 versus Con group; # *p* < 0.05, ## *p* < 0.01, ### *p* < 0.001 versus LPS group.

In order to further verify the protective effect of SA on intestinal injury in mice, this study measured the oxidative stress index of mouse ileum tissue and the level of inflammatory factors in mouse serum using a reagent kit. As shown in Figure 5A–D, compared with the Con group, the LPS group showed a significant increase in MDA and LDH content, and a significant decrease in GSH and SOD content. The levels of MDA and LDH in the SA intervention group showed a decreasing trend, while the levels of GSH and SOD showed an increasing trend. When the SA administration reached 50 mg/kg, the levels of MDA, LDH, GSH, and SOD were close to those in the Con group.

**Figure 6:**
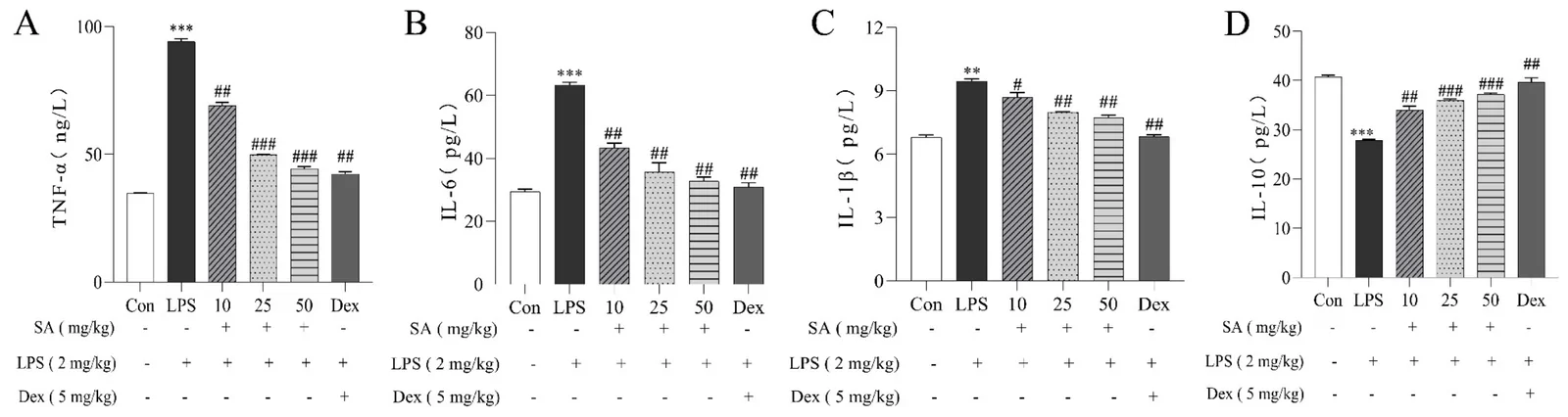
SA regulated inflammatory mediators in LPS-challenged mice. (**A**) Serum TNF-α level. (**B**) Serum IL-6 level. (**C**) Serum IL-1β level. (**D**) Serum IL-10 level. Symbols “−” and “+” indicate absence and treatment with the corresponding agent, respectively. Data are expressed as mean ± SD, *n* = 3. * *p* < 0.05, ** *p* < 0.01, *** *p* < 0.001 versus Con group; # *p* < 0.05, ## *p* < 0.01, ### *p* < 0.001 versus LPS group.

As shown in Figure 6A–D, the levels of pro-inflammatory cytokines TNF-α, IL-6, and IL-1 β increased in the LPS group, while the level of anti-inflammatory cytokine IL-10 decreased significantly, indicating a significant inflammatory response in the mice and successful modeling of intestinal injury in mice. After SA administration, the levels of pro-inflammatory factors showed a decreasing trend, while the levels of anti-inflammatory factors showed an increasing trend. When the dose reached 50 mg/kg, the levels of TNF-α, IL-6, IL-1 β, and IL-10 were close to those of the Con group.

**Figure 7:**
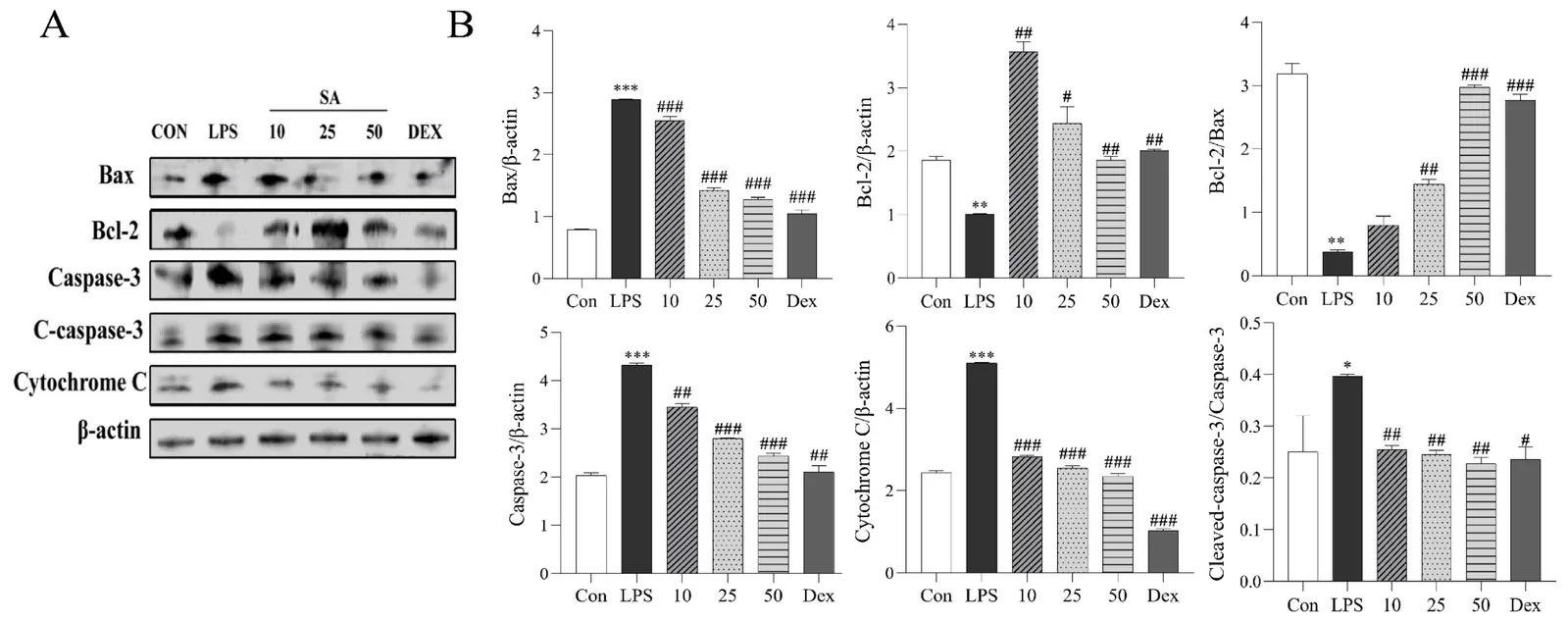
Effects of SA on apoptosis-associated proteins in the ileum of LPS-induced mice. (**A**) Western blot images of target proteins. (**B**) Densitometric quantification of the relative protein expression levels. Data are expressed as mean ± SD, *n* = 3. * *p* < 0.05, ** *p* < 0.01, *** *p* < 0.001 versus Con group; # *p* < 0.05, ## *p* < 0.01, ### *p* < 0.001 versus LPS group.

**Figure 8:**
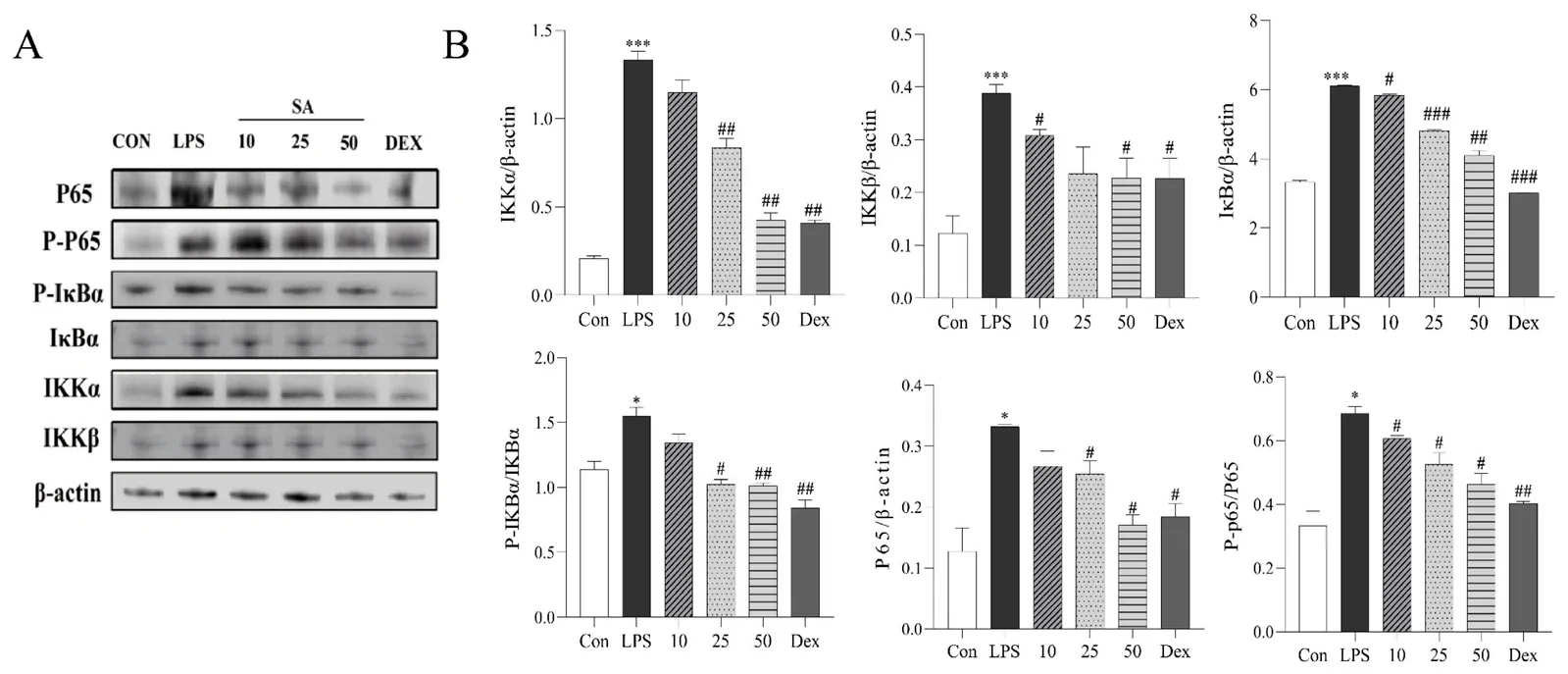
Effects of SA on the NF-κB signaling pathway in the ileum of LPS-induced mice. (**A**) Western blot images of target proteins. (**B**) Densitometric quantification of the relative protein expression levels. Data are expressed as mean ± SD, *n* = 3. * *p* < 0.05, ** *p* < 0.01, *** *p* < 0.001 versus Con group; # *p* < 0.05, ## *p* < 0.01, ### *p* < 0.001 versus LPS group.

The expression levels of apoptosis- and NF-κB pathway-related proteins in mouse ileal tissue were examined by Western blotting (Figure 7 and Figure 8). Compared with the Con group, LPS exposure triggered significant activation of pro-apoptotic signaling and the NF-κB pathway, while SA and Dex treatments significantly reversed these abnormal alterations. Collectively, these results demonstrate that SA ameliorates LPS-induced oxidative stress injury and inflammatory responses, exerting a protective effect against intestinal injury through the inhibition of apoptosis and NF-κB pathways.

### 3.4. Effect of SA on NF-κ B p65 Expression in Mouse Ileum

**Figure 9:**
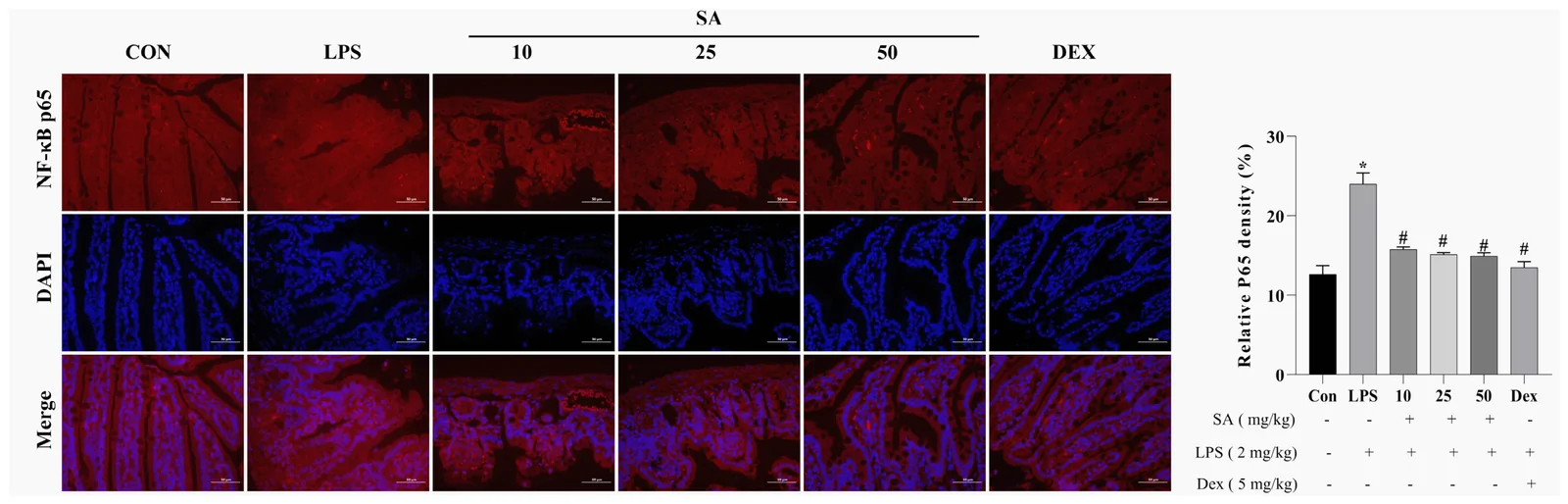
Immunofluorescence analysis of NF-κB p65 expression in colon tissues from LPS-challenged mice treated with SA (×400). Representative IF micrographs for NF-κB p65 (red), DAPI nuclear staining (blue), and merged channels, together with statistical quantification of relative NF-κB p65 density. “−”, no corresponding treatment; “+”, corresponding reagent administration. Data are mean ± SD. * *p* < 0.05 vs. Con; # *p* < 0.05 vs. LPS.

The expression of p65 protein in the mouse ileum was detected by immunofluorescence assay. Red fluorescence indicated the localization of p65 protein, while 4′,6-diamidino-2-phenylindole (DAPI) staining showed cell nuclei in blue. As shown in Figure 9, compared with the Con group, the expression of p65 protein was significantly increased in the LPS group (*p* < 0.05). After SA administration, p65 expression was inhibited to a certain extent, and the fluorescence intensity was decreased (*p* < 0.05), suggesting that SA may treat intestinal injury by reducing the expression of p65.

### 3.5. The Effect of SA on the Species Diversity of Gut Microbiota in Mice

**Figure 10:**
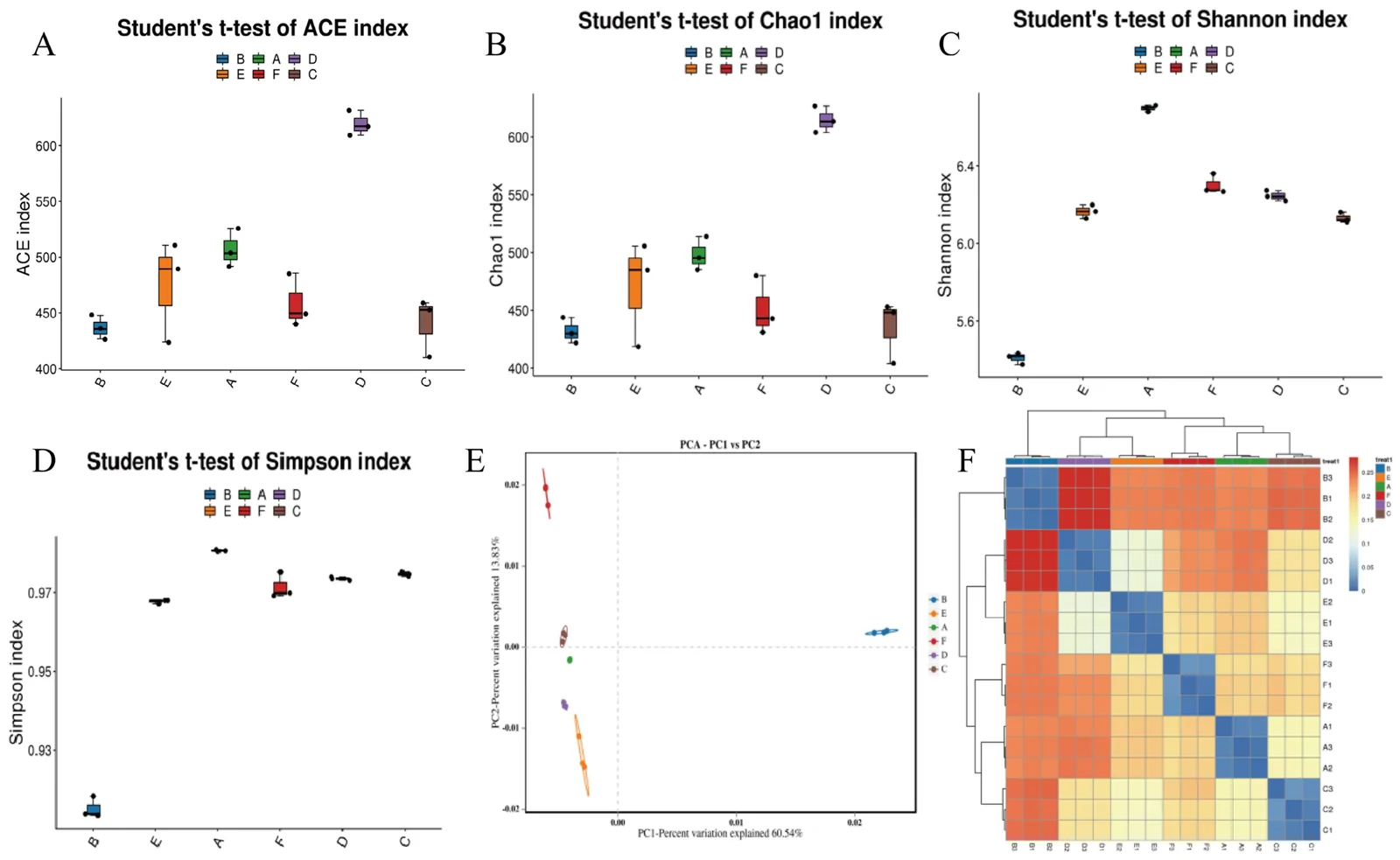
Effect of SA on species diversity of intestinal flora in mice. (**A**) ACE, (**B**) Chao 1, (**C**) Shannon, (**D**) Simpson, (**E**) PCA, (**F**) Sample Clustering Heatmap. (Note: In the figure, A: Control group; B: LPS group; C: SA 10 mg/kg group; D: SA 25 mg/kg group; E: SA 50 mg/kg group; F: Dex group).

As shown in Figure 10, alpha diversity was used to analyze the species richness and community diversity of each group. Compared with the Con group, the LPS group exhibited a disrupted gut microbiota structure, with significantly reduced Chao1 and ACE indices, as well as significantly reduced Shannon and Simpson indices. Compared with the LPS group, the SA (10, 25, 50 mg/kg) dose groups showed a significant tendency towards normalization of all these indices, indicating that SA administration reversed the LPS-induced decrease in gut microbiota species richness and community diversity, with all the above indices significantly higher than those in the LPS group. Furthermore, beta diversity was used to analyze changes in microbial community composition among groups. The principal component analysis (PCA) plot demonstrated that the points representing the Con and LPS groups were separated, while those representing the SA (10, 25, 50 mg/kg) dose groups were closer to the Con group. The sample clustering heatmap more intuitively demonstrated that the sample clustering heatmap more intuitively revealed that the gut microbial community structure of the LPS group was significantly divergent from that of the Con group and all SA treatment groups. These results indicate that LPS significantly affects gut microbial structure, while SA alleviates this effect, shifting the microbial composition towards that of the Con group.

### 3.6. Effects of SA on the Gut Microbiota Structure of Mice

**Figure 11:**
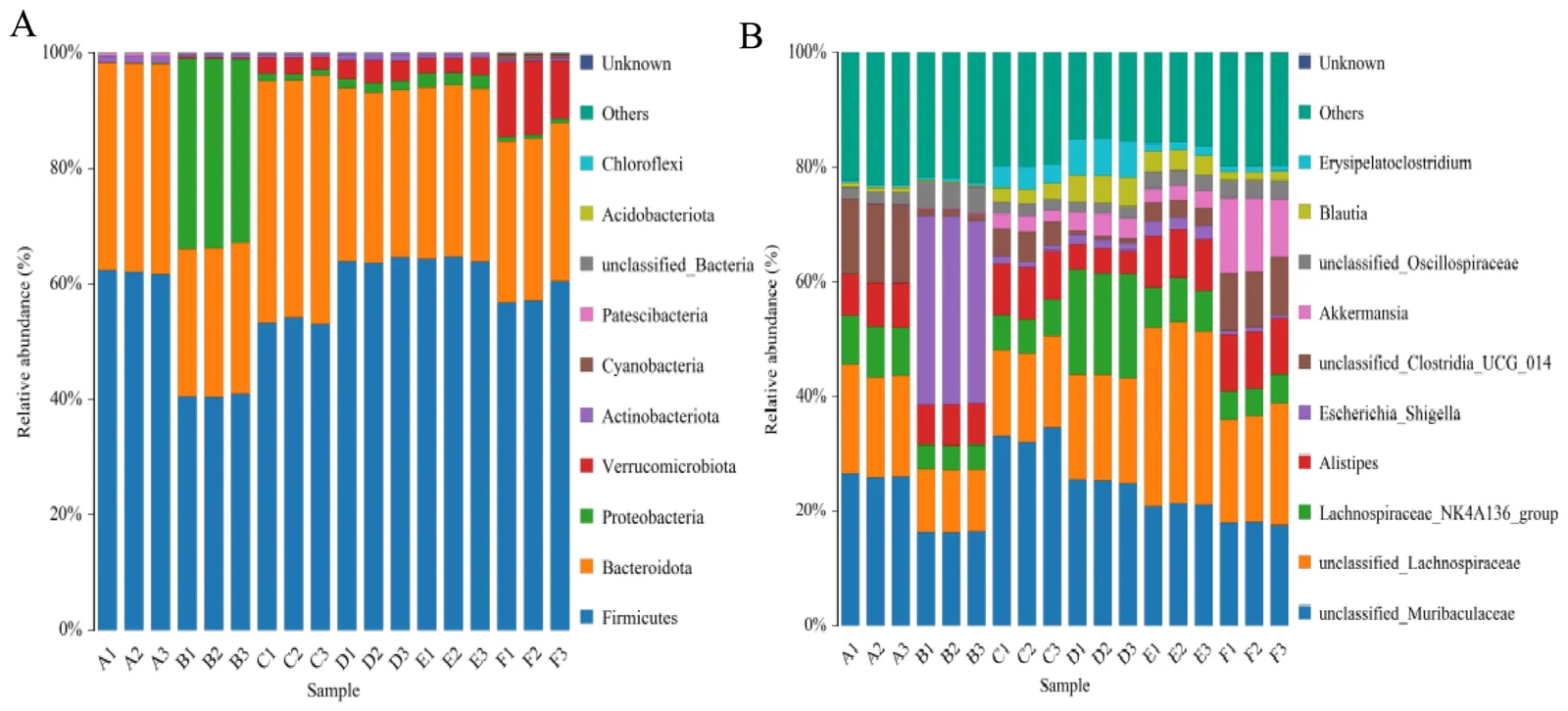
Effect of SA on the structure of intestinal flora in mice. (**A**) Phylum, (**B**) genus. (Note: In the figure, A1−A3: Control group; B1–B3: LPS group; C1−C3: SA 10 mg/kg group; D1−D3: SA 25 mg/kg group; E1−E3: SA 50 mg/kg group; F1−F3: Dex group).

As shown in Figure 11A, at the phylum level, the relative abundances of Firmicutes, Bacteroidota, and Proteobacteria changed significantly. Firmicutes and Bacteroidota were the dominant phyla in the Con group, whereas the gut microbiota of the LPS group was dominated by Proteobacteria, with markedly reduced abundances of Firmicutes and Bacteroidota. Consequently, the Firmicutes/Bacteroidota (F/B) ratio in the LPS group decreased significantly, while it was notably upregulated in the SA (50 mg/kg) dose group.

At the genus level (Figure 11B), the gut microbiota of mice in each group was primarily composed of Erysipelatoclostridium, Blautia, unclassified Oscillospiraceae, Akkermansia, unclassified Clostridia_UCG_014, Escherichia-Shigella, Alistipes, Lachnospiraceae_NK4A136_group, unclassified Lachnospiraceae, and unclassified Muribaculaceae. Escherichia-Shigella, unclassified Lachnospiraceae, and unclassified Muribaculaceae were the predominant genera, together accounting for more than half of the total composition. In contrast, the dominant genera found in the Con group were unclassified Muribaculaceae, unclassified Lachnospiraceae, and unclassified Clostridia_UCG_014. Unclassified Muribaculaceae was the most dominant genus in the SA (50 mg/kg) dose group, followed by unclassified Lachnospiraceae and Lachnospiraceae_NK4A136_group. Unclassified Lachnospiraceae was the most dominant genus in the Dex group, followed by unclassified Muribaculaceae and Akkermansia.

### 3.7. The Effect of SA on the Species Abundance of Gut Microbiota in Mice

**Figure 12:**
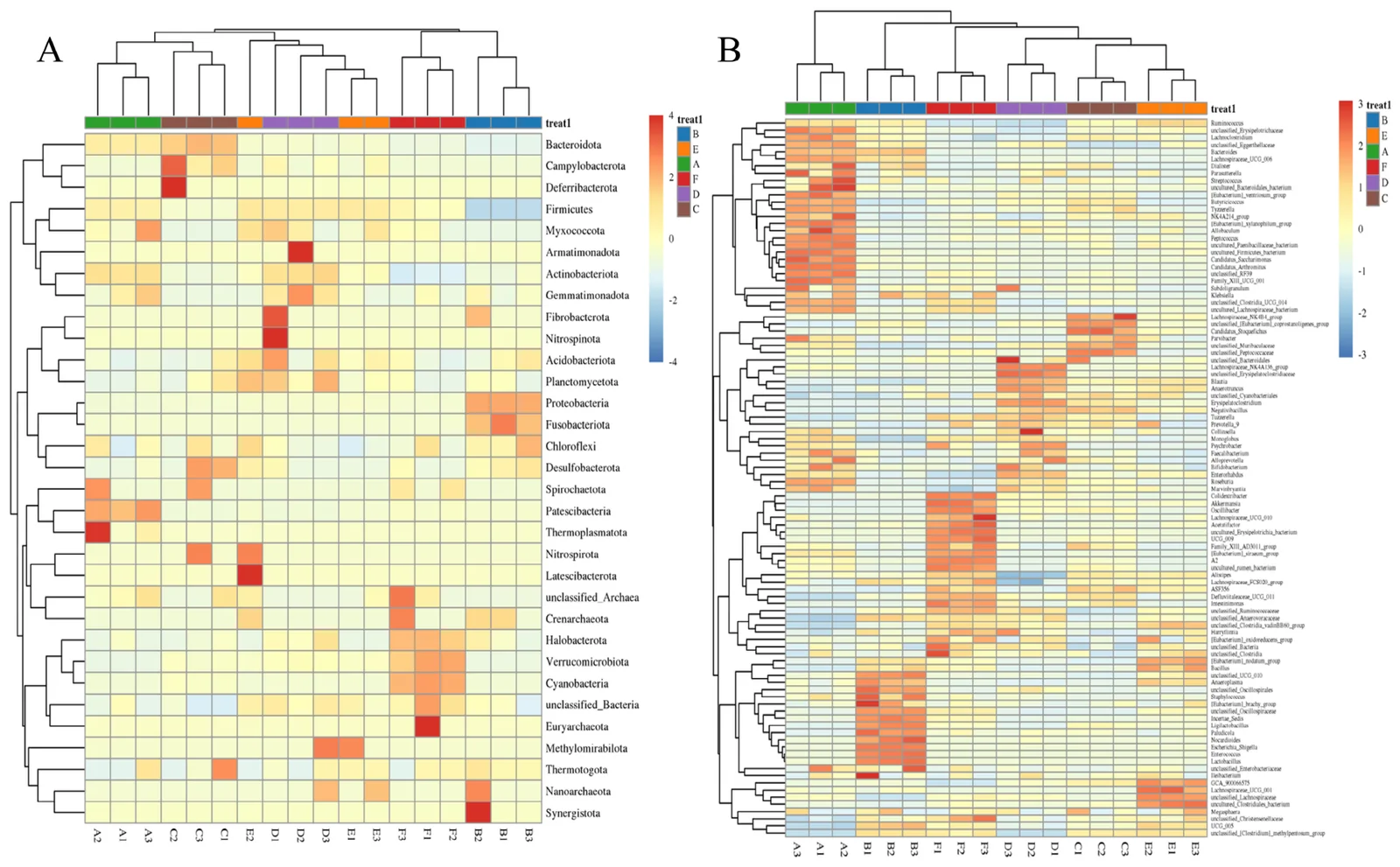
Effect of SA on the species abundance of intestinal flora in mice. (**A**) Phylum level; (**B**) genus level. The heatmap colors from blue to red indicate relative abundance (z-score) from low to high. The top colored bar represents the experimental groups (A: Control; B: LPS; C: SA 10; D: SA 25; E: SA 50; F: Dex); the left and top dendrograms represent the clustering of species and samples, respectively.

As shown in Figure 12, a visualized clustering heatmap was plotted to display the distribution and variation of gut microbiota across the Con group, LPS group, SA treatment groups (10, 25, 50 mg/kg) and Dex group among the Con group, LPS group, SA (10, 25, 50 mg/kg) dose groups, and Dex group. At the phylum level, the LPS group exhibited higher abundances of Proteobacteria and Fusobacteria, whereas the Con group was enriched in Firmicutes, Patescibacteria, Bacteroidota, and Actinobacteria.

Compared with the Con group, the LPS group exhibited significant alterations in gut microbiota composition. Specifically, the relative abundance of Parasutterella, Peptococcus, Candidatus Saccharimonas, Streptococcus, Muribaculaceae, Clostridia_UCG_014, Faecalibacterium, Candidatus Arthromitus, Roseburia, Eggertellaceae, Enterorhabdus, NK4A214_group, and Parvibacter was significantly decreased. In contrast, the relative abundance of Bacillus, UCG_010, and Oscillospiraceae was also significantly reduced, while that of Staphylococcus, Incertae Sedis, Nocardioides, Escherichia-Shigella, Enterococcus, Lactobacillus, and unclassified Enterobacteriaceae was significantly increased.

Furthermore, comparison between the LPS group and the SA groups revealed that the abundances of nine genera (unclassified UCG_010, unclassified Oscillospiraceae, Staphylococcus, Incertae_Sedis, Nocardioides, Escherichia-Shigella, Enterococcus, Lactobacillus, and unclassified Enterobacteriaceae) were significantly decreased, while the abundances of four genera (Lachnospiraceae_NK4B4_group, Candidatus_Stoquefichus, Blautia, and Lachnospiraceae_NK4A136_group) were significantly increased.

We also observed significant differences in twelve bacterial genera between the LPS group and the Dex group (Table 2). Among the different taxa, Akkermansia, Alistipes, and Lachnospiraceae_UCG_010 were more abundant in the Dex group than in the LPS group, whereas unclassified UCG_010, unclassified Oscillospiraceae, Staphylococcus, Incertae_Sedis, Nocardioides, Escherichia-Shigella, Enterococcus, Lactobacillus, and unclassified Enterobacteriaceae showed the opposite trend.

Taken together, LPS exposure led to a significant reduction in gut microbial diversity accompanied by intestinal injury. Compared with the LPS group, the principal microbial components of the SA groups were closer to those of the Con group. The SA administration reversed these alterations, particularly affecting several key functional genera, such as unclassified Muribaculaceae, Blautia, Alistipes, and Lachnospiraceae_NK4B4_group. In conclusion, SA increased the gut microbial diversity indices and significantly altered the principal microbial components in a concentration-dependent manner, restoring the LPS-induced gut microbial dysbiosis.

### 3.8. The Effect of SA on the Distribution of Gut Microbiota in Mice

**Figure 13:**
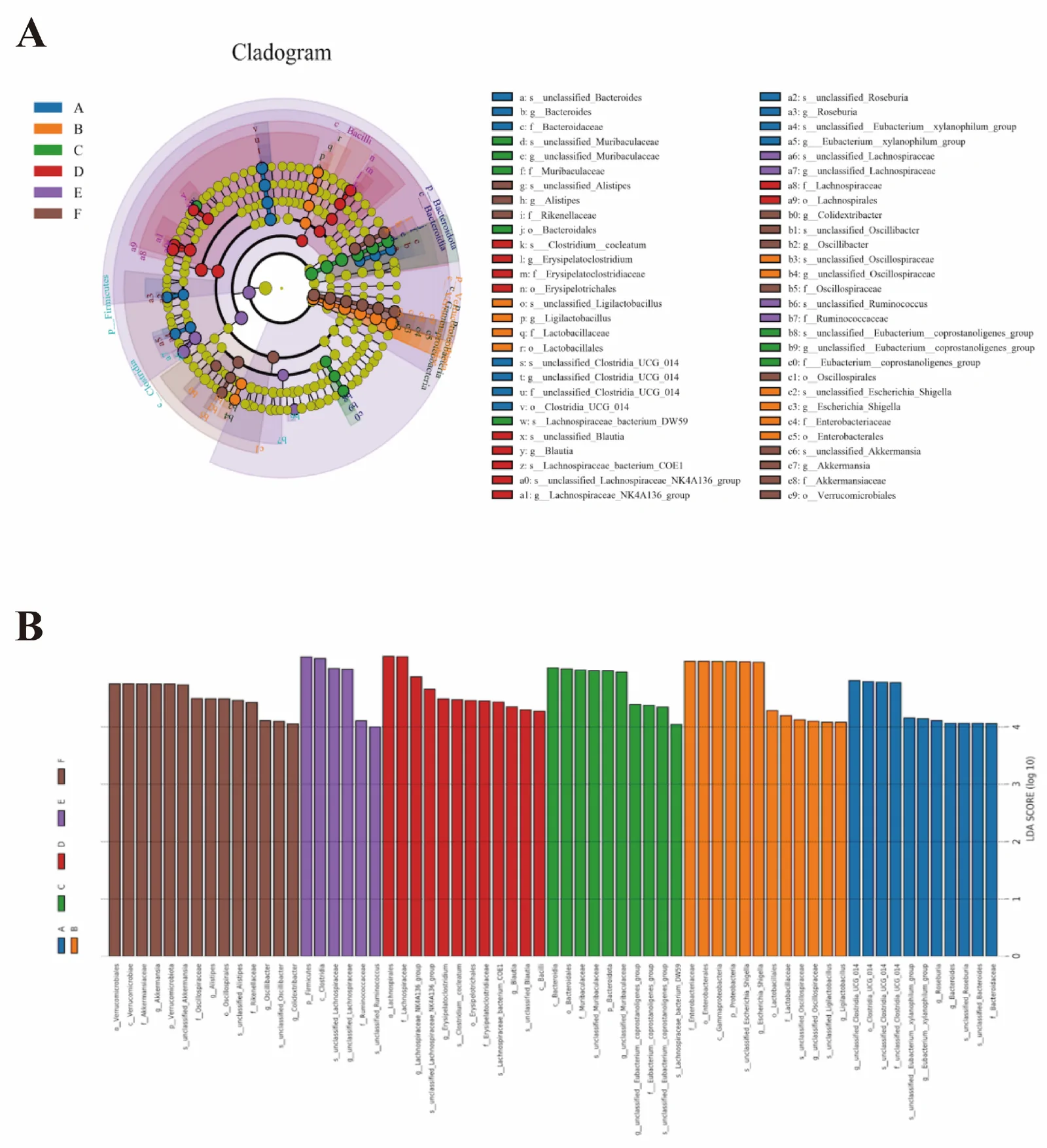
Effect of SA on the species abundance of intestinal flora in mice. (**A**) LEfSe Differential Species Annotation Map, (**B**) Differential Species Score Map. (Note: In the figure, A: Control group; B: LPS group; C: SA 10 mg/kg group; D: SA 25 mg/kg group; E: SA 50 mg/kg group; F: Dex group. In Figure 9, the histogram shows the bacterial taxa with LDA scores > 2.0. The colors of the bars correspond to the group (A–F) in which the specific taxon is most enriched).

A comprehensive analysis based on Linear discriminant analysis Effect Size (LEfSe) cladograms and differential species score plots was performed to investigate the effect of SA on the distribution of gut microbiota in mice. As illustrated in Figure 13, the LEfSe analysis revealed distinct differences in bacterial taxa among the groups. Regarding the comparison between the LPS group and the SA dose groups, the SA treatment groups showed significantly higher abundances of the Eubacterium_coprostandigenes_group, Lachnospiraceae, and Ruminococcus than the LPS group. These alterations in species abundance may be associated with the impact of SA on the intestinal environment.

### 3.9. The Effect of SA on Serum Metabolomics in Mice with Intestinal Injury

**Figure 14:**
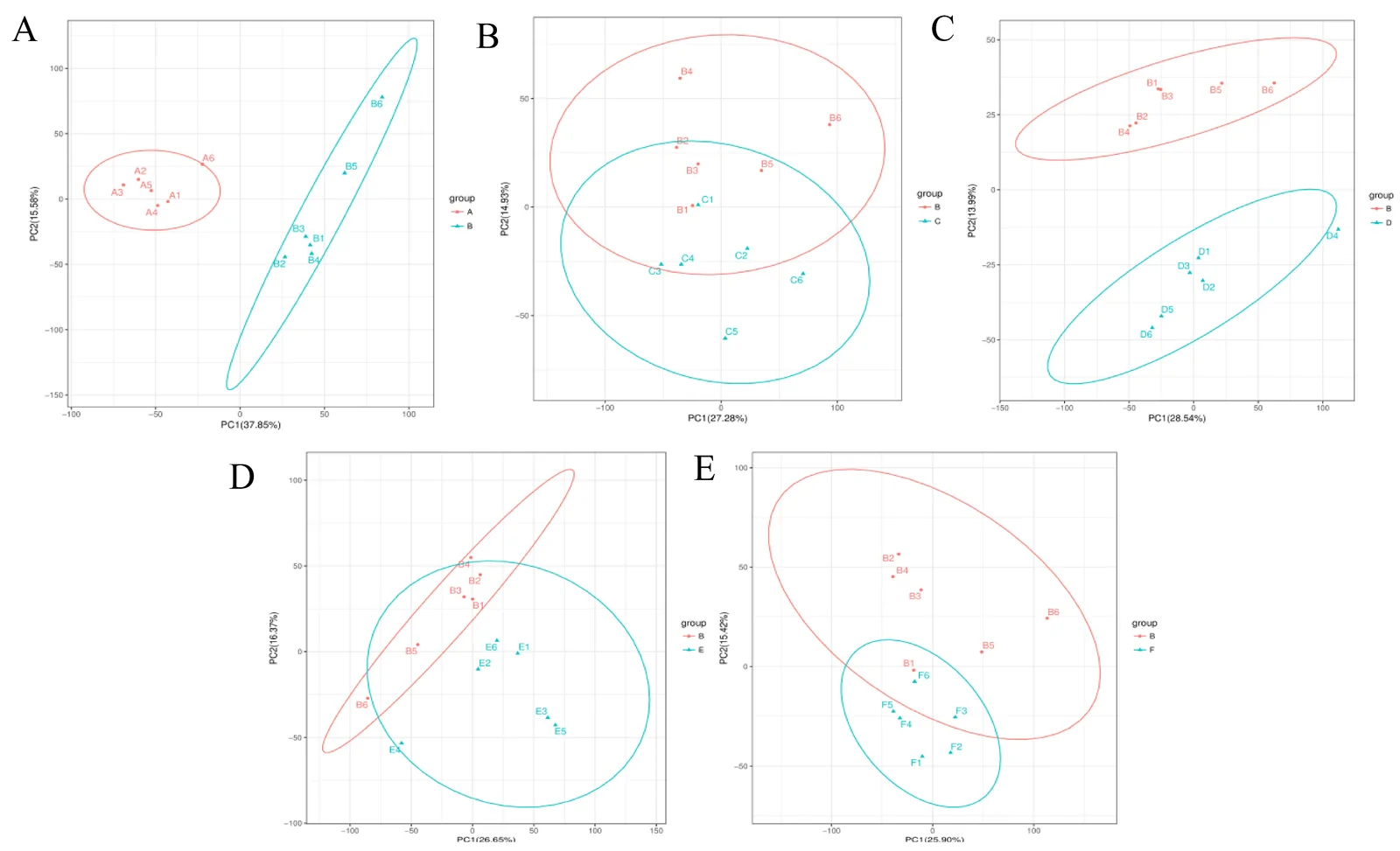
PCA score plots of serum metabolic profiles in mice from different groups. (**A**) Con vs. LPS; (**B**) LPS vs. Dex; (**C**) LPS vs. SA−10; (**D**) LPS vs. SA−25; (**E**) LPS vs. SA−50. Each point represents a biological sample, and different colors and shapes indicate different groups. The ellipses represent the 95% confidence intervals. The values in parentheses on the axes (PC1 and PC2) represent the variance explained by the respective principal components.

**Figure 15:**
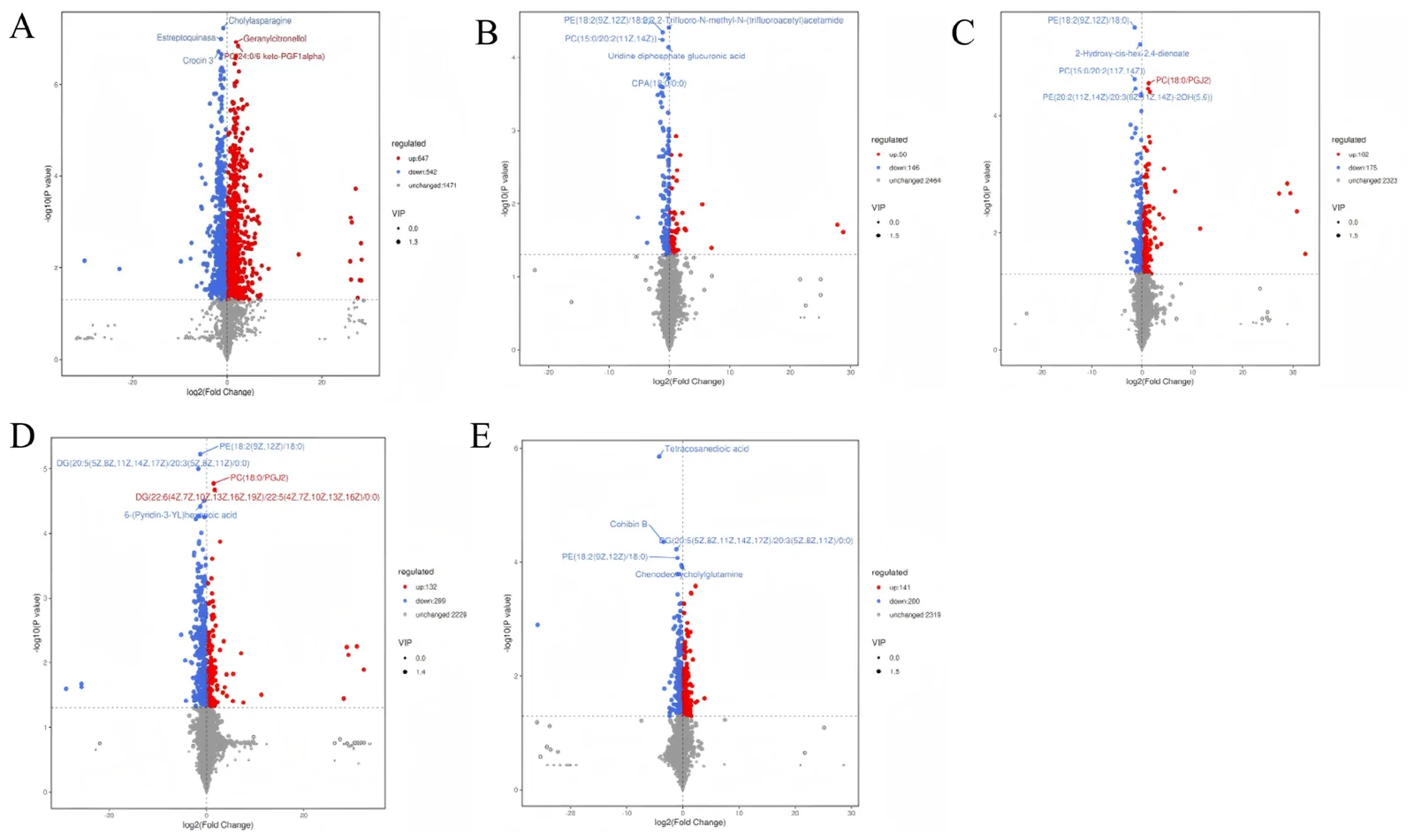
Volcano plots of differential metabolites in different groups. (**A**) Con vs. LPS; (**B**) LPS vs. Dex; (**C**) LPS vs. SA−10; (**D**) LPS vs. SA−25; (**E**) LPS vs. SA−50. Red and blue dots denote significantly upregulated and downregulated metabolites, respectively (*p* < 0.05, VIP > 1 as important markers), while gray dots indicate no significant differences.

Based on the LC-QTOF platform, the abnormal metabolic profiles in mice were detected and analyzed. As shown in Figure 14, a clear separation between the Con group and the LPS group was observed, indicating that metabolic changes occurred in the mice following LPS exposure. After intervention, the metabolic profiles of the Dex group and all SA treatment groups deviated obviously from the LPS group. The Dex group exhibited an obvious tendency toward the Con group. SA exerted regulatory effects in a dose-dependent manner; the 25 mg/kg and 50 mg/kg SA groups displayed a stronger recovery effect than the low-dose SA group. Furthermore, to identify specific perturbed metabolites, volcano plots were constructed to visualize the significantly changed metabolites among the different groups (Figure 15). Collectively, these findings demonstrated that the metabolite profiles of the LPS group were significantly distinct from those of the Con group, and both SA and Dex treatment could effectively modulate perturbed metabolite levels induced by LPS.

### 3.10. Differential Metabolite Analysis

As shown in Table 3, after merging data acquired in positive and negative ion modes, metabolite analysis was performed based on fold changes and statistical significance between groups. Differential metabolites were screened with the thresholds of *p* < 0.05, |fold change| > 1.5, and variable importance in projection (VIP) ≥ 1. Compared with the Con group, 647 metabolites were upregulated and 542 metabolites were downregulated in the LPS group.

According to VIP value and biological relevance to intestinal inflammation, we prioritized several representative metabolites. Upon LPS challenge, pro-inflammatory lipid mediators phosphatidylcholine (PC) and diacylglycerol (DG) were significantly increased. DG acts as a canonical pro-inflammatory second messenger to trigger PKC-dependent NF-κB signalling, whereas PC can be hydrolyzed by phospholipases to produce DG and other lipid mediators facilitating inflammatory activation [36]. Meanwhile, metabolites related to intestinal barrier function, including cholylasparagine and crocin 3, were markedly decreased. After SA treatment, these disordered metabolites exhibited an obvious trend toward normalization: SA intervention effectively reversed LPS-mediated elevation of PC and DG, and restored the levels of cholylasparagine and crocin 3.

Collectively, these findings suggest that dysregulated metabolites are primarily involved in amino acid and lipid serum profiles. These results lay a foundation for exploring how SA regulates serum metabolites to alleviate LPS-triggered intestinal injury in mice.

### 3.11. Metabolic Pathway Analysis of Differential Metabolites

**Figure 16:**
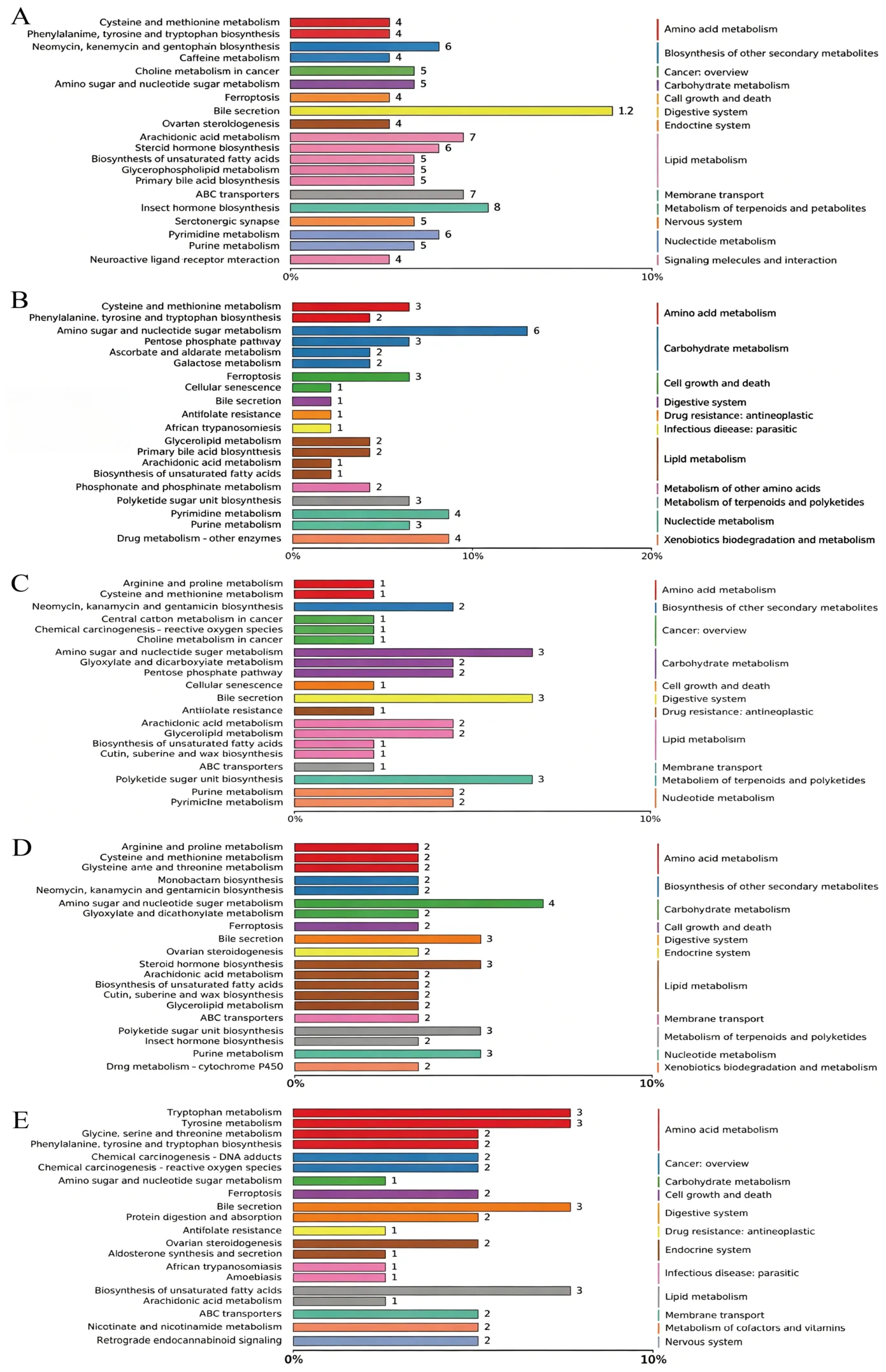
KEGG pathway classification of differential metabolites. (**A**) Con vs. LPS; (**B**) LPS vs. Dex; (**C**) LPS vs. SA−10; (**D**) LPS vs. SA−25; (**E**) LPS vs. SA−50. The x-axis indicates the percentage of annotated metabolites (%), the numbers above the bars represent the metabolite count, and the text on the right denotes the macroscopic pathway categories.

Metabolic pathway enrichment analysis of differentially expressed metabolites was performed using the Kyoto Encyclopedia of Genes and Genomes (KEGG) metabolite database, and the obtained pathways were analyzed by inter-group comparison. The vertical axis represents metabolic pathways, while the horizontal axis represents enrichment factors; the magnitude of the enrichment factor indicates the degree of metabolite enrichment in the corresponding pathway. Twenty pathways, including amino sugar and nucleotide sugar-related pathways, phenylalanine-related pathways, and glycolipid-related pathways, are key metabolic pathways involved in the SA-mediated improvement of LPS-induced intestinal injury in mice. The results are shown in Figure 16.

Collectively, LPS modeling triggered significant intestinal metabolic disorders, characterized by marked alterations in amino acid and lipid metabolites, accompanied by activation of the NF-κB-mediated inflammatory pathway and exacerbated apoptosis. SA intervention not only effectively restored the metabolic profiles but also alleviated oxidative stress injury by inhibiting the NF-κB signaling pathway and reducing intestinal epithelial cell apoptosis. These findings collectively demonstrate the multi-target protective effects of SA in ameliorating LPS-induced intestinal injury in mice.

## 4. Discussions

SA, a major bioactive lignan isolated from Schisandra chinensis, has been widely reported to possess potent anti-inflammatory and antioxidant properties in various experimental disease models [37]. Moreover, previous studies have demonstrated that certain natural compounds can alleviate intestinal barrier dysfunction by modulating apoptotic pathways and oxidative stress [38]. However, the specific protective effects of SA against acute intestinal injury induced by lipopolysaccharide (LPS), and the detailed mechanisms involving apoptosis, oxidative stress, and inflammation, remain to be fully elucidated. Therefore, the present study was designed to systematically investigate these underlying mechanisms.

A mouse model of intestinal injury was induced by LPS. Following the intraperitoneal injection of LPS, mice exhibited significant weight loss. HE staining revealed severe structural damage across the small intestine (duodenum, jileum), characterized by edema in the lamina propria, significant separation of the epithelial and lamina propria layers, widened villi, and visible serous exudation. Additionally, elevated serum DAO activity confirmed the successful establishment of the intestinal injury model.

As is well known, oxidative stress is the main cause of various diseases [39], and oxidative stress-related cell death can trigger inflammation, which is also closely associated with acute intestinal dysfunction [40,41]. In this study, LPS significantly increased the levels of MDA and LDH in small intestinal tissues while depleting the antioxidants GSH and SOD. Treatment with SA significantly attenuated these abnormalities in a dose-dependent manner. Notably, SA intervention substantially reversed the LPS-induced elevations of these oxidative markers, bringing them back close to the basal levels of the Control group. This robust antioxidant capacity of SA has been widely validated by other scholars across multiple in vitro and in vivo models. For example, SA has been reported to alleviate oxidative stress and tissue damage by activating the Nrf2/HO-1 antioxidant signaling pathway in OVA-induced allergic asthmatic mice [42]. Moreover, the effects exerted by the high-dose (50 mg/kg) SA group were comparable to those of the positive control Dex, suggesting that SA effectively alleviates oxidative stress injury in the intestinal tissue. Furthermore, consistent with the local tissue results, SA intervention significantly reversed the elevated serum levels of pro-inflammatory cytokines (TNF-α, IL-6, and IL-1β) while increasing the anti-inflammatory cytokine IL-10 (as shown in Figure 6), demonstrating that SA exerts robust anti-inflammatory effects at both the local intestinal and systemic levels.

The NF-κB signaling pathway is a pivotal regulator of inflammation, and its activation plays a crucial role in intestinal injury [43,44]. It serves as a convergence point controlling downstream cascades, including TNF-α, IL-1β, and IL-6 [45,46,47]. Elevated TNF-α triggers inflammatory cell infiltration, which directly damages surrounding intestinal tissues [48]. WB analysis showed that LPS administration significantly upregulated the expression levels of the inflammatory proteins P65, IκBα, IKKα, and IKKβ, while SA treatment significantly reduced the protein expression levels of this inflammatory pathway. This confirms that SA can reverse NF-κB pathway activation by inhibiting the expression of these proteins, thereby suppressing intestinal and systemic inflammation, which is consistent with the results of ELISA. Furthermore, SA effectively reduced the levels of the pro-inflammatory factors TNF-α, IL-1β, and IL-6, increased the level of the anti-inflammatory factor IL-10, and further downregulated NF-κB protein expression. Notably, the inhibitory efficacy achieved by the high-dose (50 mg/kg) SA group was comparable to that observed in the Dex positive control group. Thus, SA modulates mucosal inflammation and reduces intestinal permeability by suppressing LPS-induced NF-κB activation.

Exacerbated inflammatory and oxidative stress responses can lead to the accumulation of oxidative and inflammatory products, thereby inducing cell apoptosis [49,50]. Caspase-3 is a critical executioner of apoptosis, mediating cytoskeletal degradation and nuclear condensation [51,52]. Furthermore, intestinal mucosal inflammation is closely linked to the balance between the pro-apoptotic protein Bax and the anti-apoptotic protein Bcl-2 [53,54], as well as the release of Cytochrome C, which facilitates the apoptotic cascade [55]. WB results showed that SA upregulated the ratio of the anti-apoptotic protein Bcl-2 to the pro-apoptotic protein Bax, while downregulating the expression levels of Bax, caspase-3, cleaved caspase-3, and cytochrome C. These findings demonstrate that SA can effectively alleviate LPS-induced cell apoptosis. In fact, the anti-apoptotic activity of SA has been widely documented across multiple disease models. Previous research revealed that SA mitigates oxidative injury and suppresses cell apoptosis by elevating the Bcl-2/Bax ratio and decreasing cleaved caspase-3 expression in myocardial ischemia/reperfusion injury and hypoxia/reoxygenation-induced cardiomyocyte damage [56].

To further elucidate how SA improves acute intestinal barrier dysfunction, tight junction proteins were analyzed via immunohistochemistry. SA attenuated the LPS-induced degradation of ZO-1 and occludin and effectively reduced serum DAO levels, highlighting its role in maintaining functional mucosal integrity and reinforcing the intestinal barrier.

In addition, 16S rRNA sequencing revealed that LPS exposure significantly increased the abundance of certain pathogenic bacteria, including Escherichia coli, unclassified Vibrio vulnificus, and Enterococcus. Certain pathogenic strains of Escherichia coli can act as opportunistic intestinal pathogens and are closely associated with various diseases including gastritis, peritonitis, pancreatitis, and cholecystitis [57,58]. Enterococcus can also induce life-threatening pericarditis, sepsis, and meningitis; furthermore, due to inherent and acquired drug resistance, many antibiotics are ineffective in the treatment of enterococcal infections [59,60,61].

However, SA treatment reduced the abundance of these potential pathogenic bacteria, a change that is beneficial for maintaining gut microbiota homeostasis and protecting host health. Furthermore, 16S rRNA analysis revealed that SA significantly ameliorated LPS-induced alterations in gut microbial abundance and evenness, restoring them to levels similar to those in the control group (Con), and significantly reversed gut microbiota dysbiosis. These results suggest that SA may alleviate LPS-induced diarrhea in mice by reducing the proportion of harmful bacteria.

Metabolomic analyses indicated that SA exerts protective effects on the murine ileum by modulating specific metabolites and pathways. LPS profoundly disrupted intestinal metabolic profiles. Statistical filtering identified metabolites that exhibited opposing trends between the LPS and control groups. Among these, the top 10 metabolites that were restored by SA (along with those in the Dex reference group) toward control levels were selected for cluster analysis. Notably, SA upregulated phosphatidylcholine (PC) and phosphatidylglycerol (PG) in intestinal tissues. PC is critical for enterocyte function and survival, whereas PG participates in multiple protective metabolic pathways.

Kyoto Encyclopedia of Genes and Genomes (KEGG) pathway integration revealed that SA confers intestinal protection by modulating amino sugar and nucleotide sugar-related pathways, phenylalanine-related pathways, and glycolipid-related pathways. Among these, amino sugar and nucleotide sugar-associated pathways regulate crucial biochemical processes, including energy-related biochemical events and protein synthesis, thereby protecting small intestinal cells from damage [62]. SA may regulate the synthesis, degradation, or utilization of amino sugars and nucleotide sugars by modulating their associated biochemical pathways, thereby influencing small intestinal cell function. This regulatory effect may involve processes such as energy-related biochemical events and protein synthesis in small intestinal cells. By altering the profiles linked to amino sugar and nucleotide sugar pathways, SA may protect the small intestine and attenuate small intestinal cell damage. Additionally, phenylalanine-related pathways play an important role in maintaining small intestinal function and biochemical homeostasis [63]. SA regulates the biochemical status of intestinal cells, thereby reducing cell damage and inflammatory responses. Glycerolipid-associated pathways are critical for intestinal function. SA may enhance the utilization and absorption of glycerides by small intestinal cells through regulating their digestion and absorption, thereby exerting intestinal protective effects. Notably, the modulatory effects of high-dose SA (50 mg/kg) on these pathways were comparable to those of the positive control Dexamethasone (Dex) group. In this context, the comparable efficacy of high-dose SA to Dex suggests that SA may serve as a safer natural alternative with fewer side effects for treating intestinal injury, especially given that the long-term application of dexamethasone is severely restricted by well-known adverse effects, including immunosuppression, hyperglycemia, and osteoporosis [64].

## 5. Conclusions

In conclusion, our findings demonstrate that SA alleviates LPS-induced acute intestinal injury by suppressing inflammatory and oxidative stress responses, inhibiting epithelial apoptosis, and concurrently restoring gut microbial and metabolic homeostasis. These protective effects are mediated, at least in part, through the inhibition of the NF-κB signaling pathway. Collectively, these results indicate that SA holds promise as a potential nutritional supplement or functional food ingredient for the prevention and adjunctive treatment of intestinal inflammatory diseases.

However, several limitations should be acknowledged. As a pilot study, the 24 h observation period restricts our understanding to the acute phase of injury, precluding conclusions regarding long-term efficacy. Additionally, the exclusive use of male mice limits the generalizability of sex-specific responses, and the findings from animal models cannot be directly translated to clinical settings. Therefore, future studies incorporating extended observation periods, female cohorts, and prospective clinical trials are necessary to validate its clinical translational potential.

## Figures and Tables

**Table 1:** Scoring criteria for histopathological injury of the small intestine.

Score	Category	Histopathological Characteristics
0	Normal	Intact villi and crypts, no inflammatory infiltration.
1	Mild	Slight villous edema and mild epithelial shedding.
2	Moderate	Villous shortening, partial epithelial loss, and lamina propria exposure.
3	Severe	Marked villous atrophy, extensive epithelial shedding, and severe edema.
4	Very severe	Complete villous destruction, hemorrhage, or ulceration.

**Table 2:** Differential abundance of bacterial genera between the Dex group and the LPS group.

Genus	Abundance Trend (Dex Group Compared with LPS Group)	*p*-Value
*Akkermansia*	Increased	0.026
*Alistipes*	Increased	0.018
*Lachnospiraceae_UCG_010*	Increased	0.033
unclassified *UCG_010*	Decreased	0.041
unclassified *Oscillospiraceae*	Decreased	0.013
*Staphylococcus*	Decreased	0.009
*Incertae_Sedis*	Decreased	0.037
*Nocardioides*	Decreased	0.022
*Escherichia-Shigella*	Decreased	0.006
*Enterococcus*	Decreased	0.031
*Lactobacillus*	Decreased	0.016

**Table 3:** Summary of differential metabolite counts among experimental groups.

Group Comparison	Total Metabolites	Differentially Expressed (Diff.)	Upregulated	Downregulated
A vs. B (Con vs. LPS)	2660	1189	647	542
B vs. C (LPS vs. SA 10)	2660	196	50	146
B vs. D (LPS vs. SA 25)	2660	337	162	175
B vs. E (LPS vs. SA 50)	2660	431	132	299
B vs. F (LPS vs. Dex)	2660	341	141	200

Note: All statistics are calculated based on a total of 2660 metabolites detected in serum samples. The screening criteria for differential metabolites were set as *p* < 0.05, |fold change (FC)| > 1.5, and variable importance in projection (VIP) ≥ 1. Group labels: A, Control group; B, LPS group; C, SA 10 mg/kg group; D, SA 25 mg/kg group; E, SA 50 mg/kg group; F, Dex group. “Diff.” indicates the total number of differentially expressed metabolites satisfying the above screening thresholds. This table summarizes the count of differential metabolites for each comparison rather than listing individual metabolite *p*-values.

## Data Availability

Data will be made available on request.

## References

[1] Xu P., Zhang M., Cui S., Wang L., Li Y., Li Y., Jiang C. (2025). Gut microbiome and peritoneal inflammation: A new perspective on peritoneal dialysis-associated peritonitis. Ren. Fail..

[2] Liu D., Wen L., Wang Z., Hai Y., Yang D., Zhang Y., Bai M., Song B., Wang Y. (2022). The Mechanism of Lung and Intestinal Injury in Acute Pancreatitis: A Review. Front. Med..

[3] Yu C., Wang Z., Tan S., Wang Q., Zhou C., Kang X., Zhao S., Liu S., Fu H., Yu Z. (2016). Chronic Kidney Disease Induced Intestinal Mucosal Barrier Damage Associated with Intestinal Oxidative Stress Injury. Gastroenterol. Res. Pract..

[4] He S., Wang L., Wang Z., Yu S., Zhu Q., Xu Y., Jiang Y., Wu Y., Xiang H. (2025). CTSB mediates oxidative stress and intestinal epithelial barrier disruption in intestinal ischemia-reperfusion injury. Mol. Med. Rep..

[5] Barbara G., Barbaro M.R., Fuschi D., Palombo M., Falangone F., Cremon C., Marasco G., Stanghellini V. (2021). Inflammatory and Microbiota-Related Regulation of the Intestinal Epithelial Barrier. Front. Nutr..

[6] Wan C., Lu R., Zhu C., Wu H., Shen G., Yang Y., Wu X., Fang B., He Y. (2023). Ginsenoside Rb1 enhanced immunity and altered the gut microflora in mice immunized by H1N1 influenza vaccine. Peerj.

[7] Liyuan B., Jingyi T., Yirong M., Yihui L., Liping S. (2026). Effect of a Polysaccharide from Cultivated *Buchwaldoboletus* sp. on DSS-Induced Ulcerative Colitis in Mice. Nutrients.

[8] Yang K., Qiu J., Huang Z., Yu Z., Wang W., Hu H., You Y. (2021). A comprehensive review of ethnopharmacology, phytochemistry, pharmacology, and pharmacokinetics of *Schisandra chinensis* (Turcz.) Baill. and *Schisandra sphenanthera* Rehd. et Wils. J. Ethnopharmacol..

[9] Wu Y., Ding C., Liu C., Dan L., Xu H., Li X., Li Y., Song X., Zhang D. (2024). Schisandrol A, the Major Active Constitute in Schisandra chinensis: A Review of Its Preparation, Biological Activities, and Pharmacokinetics Analysis. Am. J. Chin. Med..

[10] Zhang Z., Deng Y., Feng L., Su Y., Xu D. (2022). Study on alleviate effect of Wuzhi capsule (*Schisandra sphenanthera* Rehder & E.H. Wilson extract) against mycophenolate mofetil-induced intestinal injury. J. Ethnopharmacol..

[11] Mu X., Li B., Zou Y., Liu J., Zhang B., Xiao P., Liu H. (2023). Research progress on chemical constituents of *Schisandra chinensis* and its effect on nonalcoholic fatty liver disease. China J. Chin. Mater. Medica.

[12] Wu Z., Jia M., Zhao W., Huang X., Yang X., Chen D., Qiaolongbatu X., Li X., Wu J., Qian F. (2022). Schisandrol A, the main active ingredient of Schisandrae Chinensis Fructus, inhibits pulmonary fibrosis through suppression of the TGF-β signaling pathway as revealed by UPLC-Q-TOF/MS, network pharmacology and experimental verification. J. Ethnopharmacol..

[13] Chen X., Tang R., Liu T., Dai W., Liu Q., Gong G., Song S., Hu M., Huang L., Wang Z. (2019). Physicochemical properties, antioxidant activity and immunological effects in vitro of polysaccharides from *Schisandra sphenanthera* and *Schisandra chinensis*. Int. J. Biol. Macromol..

[14] Da Silva A.S.R., Fernandes C.C., Dos Santos D.A., Mazza M.C.M., Silva J.B.A., Magalhães L.G., Pires R.H., Miranda M.L.D., Crotti A.E.M. (2024). Antileishmanial and Antifungal Activities of Volatile Oils from *Cinnamomum cassia* Bark and *Schinus molle* Leaves. Chem. Biodivers..

[15] Nowak A., Zakłos-Szyda M., Błasiak J., Nowak A., Zhang Z., Zhang B. (2019). Potential of *Schisandra chinensis* (Turcz.) Baill. in Human Health and Nutrition: A Review of Current Knowledge and Therapeutic Perspectives. Nutrients.

[16] Lee Y.M., Son E., Kim S., Kim D. (2022). Anti-Inflammatory and Analgesic Effects of *Schisandra chinensis* Leaf Extracts and Monosodium Iodoacetate-Induced Osteoarthritis in Rats and Acetic Acid-Induced Writhing in Mice. Nutrients.

[17] Xiao Z., Xiao W., Li G. (2022). Research Progress on the Pharmacological Action of Schisantherin A. Evid.-Based Complement. Altern. Med..

[18] Deng Y., Zhang Z., Hong Y., Feng L., Su Y., Xu D. (2022). Schisandrin A alleviates mycophenolic acid-induced intestinal toxicity by regulating cell apoptosis and oxidative damage. Toxicol. Mech. Methods.

[19] Yan T., Sun Y., Gong G., Li Y., Fan K., Wu B., Bi K., Jia Y. (2019). The neuroprotective effect of schisandrol A on 6-OHDA-induced PD mice may be related to PI3K/AKT and IKK/IκBα/NF-κB pathway. Exp. Gerontol..

[20] Zhou X., Zhao S., Liu T., Yao L., Zhao M., Ye X., Zhang X., Guo Q., Tu P., Zeng K. (2022). Schisandrol A protects AGEs-induced neuronal cells death by allosterically targeting ATP6V0d1 subunit of V-ATPase. Acta Pharm. Sin. B.

[21] Han S.J., Jun J., Eyun S., Lee C., Jeon J., Pan C. (2021). Schisandrol A Suppresses Catabolic Factor Expression by Blocking NF-κB Signaling in Osteoarthritis. Pharmaceuticals.

[22] Bao J., Zhang Y., Zhang L., Gong X., Shi W., Liu L., Wang X. (2021). Therapeutic effect of Schisandrin A on avian colibacillosis through gut-liver axis. Poult. Sci..

[23] Du H., Tan X., Li Z., Dong H., Su L., He Z., Ma Q., Dong S., Ramachandran M., Liu J. (2023). Effects of *Schisandra chinensis* Polysaccharide-Conjugated Selenium Nanoparticles on Intestinal Injury in Mice. Animals.

[24] Kim M.R., Cho S., Lee H.J., Kim J.Y., Nguyen U.T.T., Ha N.M., Choi K.Y., Cha K.H., Kim J., Kim W.K. (2022). Schisandrin C improves leaky gut conditions in intestinal cell monolayer, organoid, and nematode models by increasing tight junction protein expression. Phytomedicine.

[25] Geng C., Guo Y., Wang C., Cui C., Han W., Liao D., Jiang P. (2020). Comprehensive Evaluation of Lipopolysaccharide-Induced Changes in Rats Based on Metabolomics. J. Inflamm. Res..

[26] Ghosh S.S., Wang J., Yannie P.J., Ghosh S. (2020). Intestinal Barrier Dysfunction, LPS Translocation, and Disease Development. J. Endocr. Soc..

[27] Biasizzo M., Trstenjak-Prebanda M., Dolinar K., Pirkmajer S., Završnik J., Turk B., Kopitar-Jerala N. (2021). Cystatin C Deficiency Increases LPS-Induced Sepsis and NLRP3 Inflammasome Activation in Mice. Cells.

[28] Shen L., Zhou Y., Wu X., Sun Y., Xiao T., Gao Y., Wang J. (2021). TREM1 Blockade Ameliorates Lipopolysaccharide-Induced Acute Intestinal Dysfunction through Inhibiting Intestinal Apoptosis and Inflammation Response. BioMed Res. Int..

[29] Dong L., Yuan Q., Qiu G., Zhang Y., Wang H., Yu L. (2024). Protective Effects of Tea Tree Oil on Inflammatory Injury of Porcine Intestinal Epithelial Cells Induced by Lipopolysaccharide In Vitro. Animals.

[30] Pu J., Zhou X., Liu J., Hou P., Ji M. (2021). Therapeutic potential and deleterious effect of glucocorticoids on azoxymethane/dextran sulfate sodium-induced colorectal cancer in mice. Am. J. Cancer Res..

[31] Miao F., Shan C., Shah S.A.H., Akhtar R.W., Geng S., Ning D., Wang X. (2020). The protective effect of walnut oil on lipopolysaccharide–induced acute intestinal injury in mice. Food Sci. Nutr..

[32] Shen G., Cheng D., Karunathilaka J.C., Woo J., Li D., Wooton J.L., Jaynes P., Ju T., Wang W. (2026). Watercress Extract Reduces Experimental Colitis by Modulating Inflammation and Regulating the Gut Microbiota. Nutrients.

[33] Woo J., Cheng D., Long E.A., Whitney K.L., Shen G., Reddivari L., Jiang Q., Simsek S., Ju T., Wang W. (2026). Hemp seed mitigates colonic inflammation through macrophage polarization and microbiota-barrier axis restoration. Food Funct..

[34] Qiang T., Wang J., Jiang L., Xiong K. (2022). Modulation of hyperglycemia by sodium alginate is associated with changes of serum metabolite and gut microbiota in mice. Carbohydr. Polym..

[35] Luk G.D., Bayless T.M., Baylin S.B. (1980). Diamine oxidase (histaminase). A circulating marker for rat intestinal mucosal maturation and integrity. J. Clin. Investig..

[36] Eurtivong C., Leung E., Sharma N., Leung I.K.H., Reynisson J. (2023). Phosphatidylcholine-Specific Phospholipase C as a Promising Drug Target. Molecules.

[37] Fu K., Zhou H., Wang C., Gong L., Ma C., Zhang Y., Li Y. (2022). A review: Pharmacology and pharmacokinetics of Schisandrin A. Phytother. Res..

[38] Xu Q., Liu M., Chao X., Zhang C., Yang H., Chen J., Zhou B. (2023). Stevioside Improves Antioxidant Capacity and Intestinal Barrier Function while Attenuating Inflammation and Apoptosis by Regulating the NF-κB/MAPK Pathways in Diquat-Induced Oxidative Stress of IPEC-J2 Cells. Antioxidants.

[39] Liguori I., Russo G., Curcio F., Bulli G., Aran L., Della-Morte D., Gargiulo G., Testa G., Cacciatore F., Bonaduce D. (2018). Oxidative stress, aging, and diseases. Clin. Interv. Aging.

[40] Muro P., Zhang L., Li S., Zhao Z., Jin T., Mao F., Mao Z. (2024). The emerging role of oxidative stress in inflammatory bowel disease. Front. Endocrinol..

[41] Almousa A.A., Meurens F., Krol E.S., Alcorn J. (2018). Linoorbitides and enterolactone mitigate inflammation-induced oxidative stress and loss of intestinal epithelial barrier integrity. Int. Immunopharmacol..

[42] Dong Y., Zou Y., Li T., Sun J., Li H., Zhuang W., Song Y., Wang C. (2024). Schisandrol A Alleviates Allergic Asthma in Mice via Regulating the NF-κB/IκBα and Nrf2/HO-1 Signaling Pathways. J. Med. Food..

[43] Atreya I., Atreya R., Neurath M.F. (2008). NF-kappaB in inflammatory bowel disease. J. Intern. Med..

[44] Mukherjee T., Kumar N., Chawla M., Philpott D.J., Basak S. (2024). The NF-κB signaling system in the immunopathogenesis of inflammatory bowel disease. Sci. Signal..

[45] Fujita K., Tokuda H., Yamamoto N., Kainuma S., Kawabata T., Sakai G., Kuroyanagi G., Matsushima-Nishiwaki R., Harada A., Kozawa O. (2017). Incretins amplify TNF-α-stimulated IL-6 synthesis in osteoblasts: Suppression of the IκB/NF-κB pathway. Int. J. Mol. Med..

[46] Huang L., Tang Y., Qin J., Peng Y., Yuan Q., Zhang F., Tao L. (2012). Vasoactive intestinal peptide enhances TNF-α-induced IL-6 and IL-8 synthesis in human proximal renal tubular epithelial cells by NF-κB-dependent mechanism. Inflammation.

[47] Lee I., Hyun Y., Kim D. (2010). Berberine ameliorates TNBS-induced colitis by inhibiting lipid peroxidation, enterobacterial growth and NF-κB activation. Eur. J. Pharmacol..

[48] Zhong J., Yu R., Zhou Q., Liu P., Liu Z., Bian Y. (2021). Naringenin prevents TNF-α-induced gut-vascular barrier disruption associated with inhibiting the NF-κB-mediated MLCK/p-MLC and NLRP3 pathways. Food Funct..

[49] Levy A., Stedman A., Deutsch E., Donnadieu F., Virgin H.W., Sansonetti P.J., Nigro G. (2020). Innate immune receptor NOD2 mediates LGR5+ intestinal stem cell protection against ROS cytotoxicity via mitophagy stimulation. Proc. Natl. Acad. Sci. USA.

[50] Zhao M., Wang S., Zuo A., Zhang J., Wen W., Jiang W., Chen H., Liang D., Sun J., Wang M. (2021). HIF-1α/JMJD1A signaling regulates inflammation and oxidative stress following hyperglycemia and hypoxia-induced vascular cell injury. Cell. Mol. Biol. Lett..

[51] Suria H., Chau L.A., Negrou E., Kelvin D.J., Madrenas J. (2000). Cytoskeletal disruption induces T cell apoptosis by a caspase-3 mediated mechanism. Life Sci..

[52] Li Y., Liu W., Yang W., Zhang X., Wen S., Liu K. (2015). Mo2025 miR-378 Attenuates Intestinal Ischemia/Reperfusion Injury by Inhibiting Intestinal Mucosal Apoptosis via Targeting Caspase-3 Signaling. Gastroenterology.

[53] Li Z., Gao Y., Du L., Yuan Y., Huang W., Fu X., Huang Y., Zhang X., You F., Li S. (2022). Anti-inflammatory and anti-apoptotic effects of Shaoyao decoction on X-ray radiation-induced enteritis of C57BL/6 mice. J. Ethnopharmacol..

[54] Yang J., Chen W., Sun Y., Xia P., Liu J., Zhang W. (2021). The role of microRNAs in regulating cadmium-induced apoptosis by targeting Bcl-2 in IEC-6 cells. Toxicol. Appl. Pharmacol..

[55] Chimenti M.S., Sunzini F., Fiorucci L., Botti E., Fonti G.L., Conigliaro P., Triggianese P., Costa L., Caso F., Giunta A. (2018). Potential Role of Cytochrome c and Tryptase in Psoriasis and Psoriatic Arthritis Pathogenesis: Focus on Resistance to Apoptosis and Oxidative Stress. Front. Immunol..

[56] Gong S., Liu J., Wan S., Yang W., Zhang Y., Yu B., Li F., Kou J. (2021). Schisandrol A Attenuates Myocardial Ischemia/Reperfusion-Induced Myocardial Apoptosis through Upregulation of 14-3-3θ. Oxid. Med. Cell. Longev..

[57] Yan X., Bai L., Lv J., Qi P., Song X., Zhang L. (2024). Effects of Combined Live *Bifidobacterium, Lactobacillus, Enterococcus*, and *Bacillus cereus* Tablets on the Structure and Function of the Intestinal Flora in Rabbits Undergoing Hepatic Artery Infusion Chemotherapy. Biology.

[58] Zheng J., Lou L., Fan J., Huang C., Mei Q., Wu J., Guo Y., Lu Y., Wang X., Zeng Y. (2019). Commensal *Escherichia coli* Aggravates Acute Necrotizing Pancreatitis through Targeting of Intestinal Epithelial Cells. Appl. Environ. Microbiol..

[59] Krawczyk B., Wityk P., Gałęcka M., Michalik M. (2021). The Many Faces of *Enterococcus* spp.—Commensal, Probiotic and Opportunistic Pathogen. Microorganisms.

[60] Arias C.A., Murray B.E. (2012). The rise of the *Enterococcus*: Beyond vancomycin resistance. Nat. Rev. Microbiol..

[61] Hollenbeck B.L., Rice L.B. (2012). Intrinsic and acquired resistance mechanisms in *Enterococcus*. Virulence.

[62] Li J., Jia S., Yuan C., Yu B., Zhang Z., Zhao M., Liu P., Li X., Cui B. (2022). Jerusalem artichoke inulin supplementation ameliorates hepatic lipid metabolism in type 2 diabetes mellitus mice by modulating the gut microbiota and fecal metabolome. Food Funct..

[63] Salau V.F., Erukainure O.L., Koorbanally N.A., Islam M.S. (2020). Kolaviron modulates dysregulated metabolism in oxidative pancreatic injury and inhibits intestinal glucose absorption with concomitant stimulation of muscle glucose uptake. Arch. Physiol. Biochem..

[64] Caplan A., Fett N., Rosenbach M., Werth V.P., Micheletti R.G. (2016). Prevention and management of glucocorticoid-induced side effects: A comprehensive review: Gastrointestinal and endocrinologic side effects. J. Am. Acad. Dermatol..

